# Eukaryotic virus composition can predict the efficiency of carbon export in the global ocean

**DOI:** 10.1016/j.isci.2020.102002

**Published:** 2020-12-29

**Authors:** Hiroto Kaneko, Romain Blanc-Mathieu, Hisashi Endo, Samuel Chaffron, Tom O. Delmont, Morgan Gaia, Nicolas Henry, Rodrigo Hernández-Velázquez, Canh Hao Nguyen, Hiroshi Mamitsuka, Patrick Forterre, Olivier Jaillon, Colomban de Vargas, Matthew B. Sullivan, Curtis A. Suttle, Lionel Guidi, Hiroyuki Ogata

**Affiliations:** 1 Bioinformatics Center, Institute for Chemical Research, Kyoto University, Gokasho, Uji, Kyoto 611-0011, Japan; 2 Laboratoire de Physiologie Cellulaire & Végétale, CEA, Univ. Grenoble Alpes, CNRS, INRA, IRIG, Grenoble, France; 3 Université de Nantes, CNRS UMR 6004, LS2N, 44000 Nantes, France; 4 Research Federation (FR2022) Tara Oceans GO-SEE, Paris, France; 5 Génomique Métabolique, Genoscope, Institut François Jacob, CEA, CNRS, 91000 Evry, France; 6 Sorbonne Universités, CNRS, Laboratoire Adaptation et Diversité en Milieu Marin, Station Biologique de Roscoff, 29680 Roscoff, France; 7 Institut Pasteur, Department of Microbiology, 25 rue du Docteur Roux, 75015, Paris, France; 8 Department of Microbiology and Department of Civil, Environmental and Geodetic Engineering, Ohio State University, Columbus, OH, United States of America; 9 Departments of Earth, Ocean & Atmospheric Sciences, Microbiology & Immunology, and Botany, and the Institute for the Oceans and Fisheries, University of British Columbia, Vancouver, BC V6T 1Z4, Canada; 10 Sorbonne Université, CNRS, Laboratoire d’Océanographie de Villefanche, LOV, 06230 Villefranche-sur-mer, France

**Keywords:** Oceanography, Biogeoscience, Global Carbon Cycle, Virology, Viral Microbiology, Carbon Cycle

## Abstract

The biological carbon pump, in which carbon fixed by photosynthesis is exported to the deep ocean through sinking, is a major process in Earth's carbon cycle. The proportion of primary production that is exported is termed the carbon export efficiency (CEE). Based on in-lab or regional scale observations, viruses were previously suggested to affect the CEE (i.e., viral “shunt” and “shuttle”). In this study, we tested associations between viral community composition and CEE measured at a global scale. A regression model based on relative abundance of viral marker genes explained 67% of the variation in CEE. Viruses with high importance in the model were predicted to infect ecologically important hosts. These results are consistent with the view that the viral shunt and shuttle functions at a large scale and further imply that viruses likely act in this process in a way dependent on their hosts and ecosystem dynamics.

## Highlights

• Eukaryotic virus community composition is shown to predict carbon export efficiency
• Tens of viruses are highly important in the prediction of the efficiency
• These viruses are inferred to infect ecologically important hosts

## Graphical abstract

**Figure:**
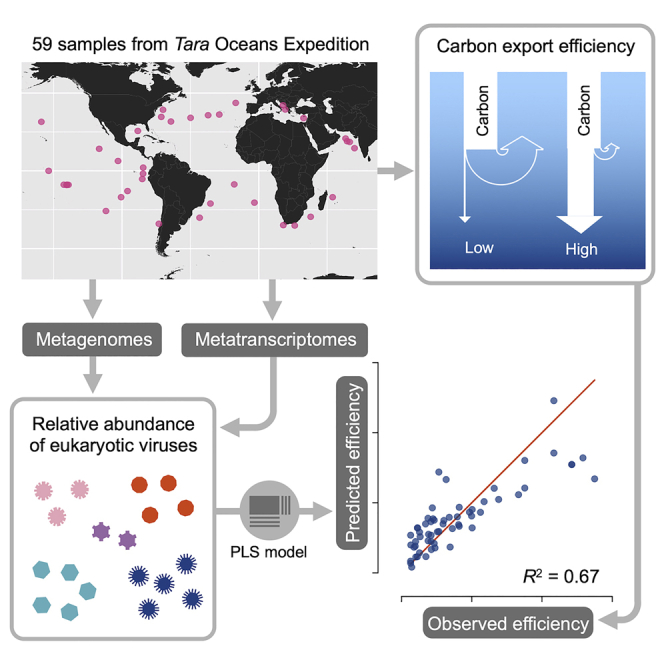

## Teaser

Oceanography; Biogeoscience; Global Carbon Cycle; Virology; Viral Microbiology; Biogeoscience; Carbon Cycle

## Introduction

A major process in the global cycling of carbon is the oceanic biological carbon pump (BCP), an organism-driven process by which atmospheric carbon (i.e., CO_2_) is transferred and sequestered to the ocean interior and seafloor for periods ranging from centuries to hundreds of millions of years. Between 15% and 20% of net primary production (NPP) is exported out of the euphotic zone, with 0.3% of fixed carbon reaching the seafloor annually (Zhang et al., 2018). However, there is wide variation in estimates of the proportion of primary production in the surface ocean that is exported to depth, ranging from 1% in the tropical Pacific to 35%–45% during the North Atlantic bloom (Buesseler and Boyd, 2009). As outlined below, many factors affect the BCP.

Of planktonic organisms living in the upper layer of the ocean, diatoms (Tréguer et al., 2018) and zooplankton (Turner, 2015) have been identified as important contributors to the BCP in nutrient-replete oceanic regions. In the oligotrophic ocean, cyanobacteria, collodarians (Lomas and Moran, 2011), diatoms (Agusti et al., 2015; Karl et al., 2012; Leblanc et al., 2018), and other small (pico-to nano-) plankton (Lomas and Moran, 2011) have been implicated in the BCP. Sediment trap studies suggest that ballasted aggregates of plankton with biogenic minerals contribute to carbon export to the deep sea (Iversen and Ploug, 2010; Klaas and Archer, 2002). The BCP comprises three processes: carbon fixation, export, and remineralization. As these processes are governed by complex interactions between numerous members of planktonic communities (Zhang et al., 2018), the BCP is expected to involve various organisms, including viruses (Zimmerman et al., 2019).

Viruses have been suggested to regulate the efficiency of the BCP. Lysis of host cells by viruses releases cellular material in the form of dissolved organic matter (DOM), which fuels the microbial loop and enhances respiration and secondary production (Gobler et al., 1997; Weitz et al., 2015). This process, coined “viral shunt (Wilhelm and Suttle, 1999),” can reduce the carbon export efficiency (CEE) because it increases the retention of nutrients and carbon in the euphotic zone and prevents their transfer to higher trophic levels as well as their export from the euphotic zone to the deep sea (Fuhrman, 1999; Weitz et al., 2015). However, an alternative process is also considered, in which viruses contribute to the vertical carbon export (Weinbauer, 2004). For instance, a theoretical study proposed that the CEE increases if viral lysis augments the ratio of exported carbon relative to the primary production-limiting nutrients (nitrogen and phosphorous) (Suttle, 2007). Laboratory experimental studies reported that cells infected with viruses form larger particles (Peduzzi and Weinbauer, 1993; Yamada et al., 2018), can sink faster (Lawrence and Suttle, 2004), and can lead to preferential grazing by heterotrophic protists (Evans and Wilson, 2008) and/or to higher growth of grazers (Goode et al., 2019). This process termed “viral shuttle” (Sullivan et al., 2017) is supported by several field studies that reported association of viruses with sinking material. Viruses were observed in sinking material in the North Atlantic Ocean (Proctor and Fuhrman, 1991) and sediment of coastal waters where algal blooms occur (Lawrence et al., 2002; Tomaru et al., 2007, 2011). In addition, vertical transport of bacterial viruses between photic and aphotic zones was observed in the Pacific Ocean (Hurwitz et al., 2015) and in *Tara* Oceans virome data (Brum et al., 2015). A systematic analysis of large-scale omics data from oligotrophic oceanic regions revealed a positive association between the magnitude of carbon flux and bacterial dsDNA viruses (i.e., cyanophages), which were previously unrecognized as possible contributors to the BCP (Guidi et al., 2016).

More recently, viral infection of blooms of the photosynthetic eukaryote *Emiliania huxleyi* in the North Atlantic were found to be accompanied by particle aggregation and greater downward vertical flux of carbon, with the highest export during the early stage of viral infection (Laber et al., 2018; Sheyn et al., 2018). Given the significant contributions of eukaryotic plankton to ocean biomass and net production (Hirata et al., 2011; Li, 1995) and their observed predominance over prokaryotes in sinking materials of Sargasso Sea oligotrophic surface waters (Fawcett et al., 2011; Lomas and Moran, 2011), various lineages of eukaryotic viruses may be responsible for a substantial part of the variation in carbon export across oceanic regions.

If the “viral shunt” and “shuttle” processes function at a global scale and if these involve specific eukaryotic viruses, we expect to detect a statistical association between eukaryotic viral community composition and CEE in a large-scale omics data. To our knowledge, such an association has never been investigated. Although this test per se does not prove that viruses regulate CEE, we consider the association is worth being tested because such an association is a necessary condition for the global model of viral shunt and shuttle and, under its absence, we would have to reconsider the model. Deep sequencing of planktonic community DNA and RNA, as carried out in *Tara* Oceans, has enabled the identification of marker genes of major viral groups infecting eukaryotes (Hingamp et al., 2013; Carradec et al., 2018; Culley, 2018; Endo et al., 2020). To examine the association between viral community composition and CEE, we thus used the comprehensive organismal dataset from the *Tara* Oceans expedition (Carradec et al., 2018; Sunagawa et al., 2015), as well as related measurements of carbon export estimated from particle concentrations and size distributions observed in situ (Guidi et al., 2016).

In the present study, we identified several hundred marker-gene sequences of nucleocytoplasmic large DNA viruses (NCLDVs) in metagenomes of 0.2–3 μm size fraction. We also identified RNA and ssDNA viruses in metatranscriptomes of four eukaryotic size fractions spanning 0.8 to 2,000 μm. The resulting profiles of viral distributions were compared with an image-based measure of carbon export efficiency (CEE), which is defined as the ratio of the carbon flux at depth to the carbon flux at surface.

## Results and discussion

### Detection of diverse eukaryotic viruses in *Tara* Oceans gene catalogs

We used profile hidden Markov model-based homology searches to identify marker-gene sequences of eukaryotic viruses in two ocean gene catalogs. These catalogs were previously constructed from environmental shotgun sequence data of samples collected during the *Tara* Oceans expedition. The first catalog, the Ocean Microbial Reference Gene Catalog (OM-RGC), contains 40 million non-redundant genes predicted from the assemblies of *Tara* Oceans viral and microbial metagenomes (Sunagawa et al., 2015). We searched this catalog for NCLDV DNA polymerase family B (PolB) genes, as dsDNA viruses may be present in microbial metagenomes because large virions (>0.2 μm) have been retained on the filter or because viral genomes actively replicating or latent within picoeukaryotic cells have been captured. The second gene catalog, the Marine Atlas of *Tara* Oceans Unigenes (MATOU), contains 116 million non-redundant genes derived from metatranscriptomes of single-cell microeukaryotes and small multicellular zooplankton (Carradec et al., 2018). We searched this catalog for NCLDV PolB genes, RNA-dependent RNA polymerase (RdRP) genes of RNA viruses, and replication-associated protein (Rep) genes of ssDNA viruses, because transcripts of viruses actively infecting their hosts, as well as genomes of RNA viruses, have been captured in this catalog.

**Figure 1:**
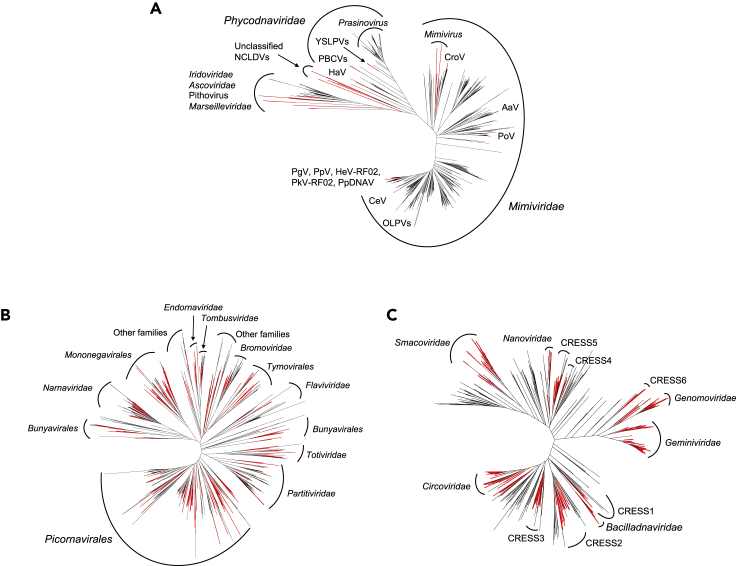
Viruses of eukaryotic plankton identified in *Tara* Oceans samples are distantly related to characterized viruses Unrooted maximum likelihood phylogenetic trees containing environmental (black) and reference (red) viral sequences for NCLDV DNA polymerase family B (A), RNA virus RNA-dependent RNA polymerase (B), and ssDNA virus replication-associated protein (C). See also Figures S1–S4

We identified 3,874 NCLDV PolB sequences (3,486 in metagenomes and 388 in metatranscriptomes), 975 RNA virus RdRP sequences, and 299 ssDNA virus Rep sequences (Table 1). These sequences correspond to operational taxonomic units (OTUs) at a 95% identity threshold. All except 17 of the NCLDV PolBs from metagenomes were assigned to the families *Mimiviridae* (*n* = 2,923), *Phycodnaviridae* (*n* = 348), and *Iridoviridae* (*n* = 198) (Table 1). The larger numbers of PolB sequences assigned to *Mimiviridae* and *Phycodnaviridae* compared with other NCLDV families are consistent with previous observations (Endo et al., 2020; Hingamp et al., 2013). The divergence between these environmental sequences and reference sequences from known viral genomes was greater in *Mimiviridae* than in *Phycodnaviridae* (Figures 1A, S1A, and S2). Within *Mimiviridae,* 83% of the sequences were most similar to those from algae-infecting *Mimivirus* relatives. Among the sequences classified in *Phycodnaviridae,* 93% were most similar to those in *Prasinovirus*, whereas 6% were closest to *Yellowstone lake phycodnavirus*, which is closely related to *Prasinovirus*. Prasinoviruses are possibly overrepresented in the metagenomes because the 0.2 to 3 μm size fraction selects their picoeukaryotic hosts. RdRP sequences were assigned mostly to the order *Picornavirales* (*n* = 325), followed by the families *Partitiviridae* (*n* = 131), *Narnaviridae* (*n* = 95), *Tombusviridae* (*n* = 45), and *Virgaviridae* (*n* = 33) (Table 1), with most sequences being distant (30%–40% amino acid identity) from reference viruses (Figures 1B, S1B, and S3). These results are consistent with previous studies on the diversity of marine RNA viruses, in which RNA virus sequences were found to correspond to diverse positive-polarity ssRNA and dsRNA viruses distantly related to well-characterized viruses (Culley, 2018). *Picornavirales* may be overrepresented in the metatranscriptomes because of the polyadenylated RNA selection. The majority (*n* = 201) of Rep sequences were annotated as *Circoviridae*, known to infect animals, which is consistent with a previous report (Wang et al., 2018). Only eight were annotated as plant ssDNA viruses (families *Nanoviridae* and *Geminiviridae*) (Table 1). Most of these environmental sequences are distant (40% to 50% amino acid identity) from reference sequences (Figures 1C, S1C, and S4). Additional 388 NCLDV PolBs were detected in the metatranscriptomes. The average cosmopolitanism (number of samples where an OTU was observed by at least two reads) for PolBs in metagenomes was 23 samples against 2.9 for metatranscriptome-derived PolB sequences, 5.5 for Reps, and 5.8 for RdRPs. Within metatranscriptomes, the average gene-length normalized read counts for PolBs were respectively ten and three times lower than those of RdRPs and Reps. Therefore, PolBs from metatranscriptomes were not further used in our study.

**Table 1:** Taxonomic breakdown of viral marker genes

Viruses		Identified	Used in PLS regression^a^
NCLDVs	Mimiviridae	2,923	1,148
	Phycodnaviridae	348	99
	Iridoviridae	198	59
	Other NCLDVs^b^	17	3
	Total	3,486	1,309
RNA viruses	Picornavirales (ssRNA+)	325	80
	Partitiviridae (dsRNA)	131	22
	Narnaviridae (ssRNA+)	95	6
	Other families	289	53
	Unclassified	78	9
	RNA viruses	57	10
	Total	975	180
ssDNA viruses	Circoviridae	201	22
	Geminiviridae	4	0
	Nanoviridae	4	0
	Unclassified	39	2
	ssDNA viruses	51	10
	Total	299	34
All		4,760	1,523

a The marker genes had to occur in at least five samples and harbor a Spearman correlation coefficient > |0.2| with carbon export efficiency.

b There was no unclassified NCLDV.

### Composition of eukaryotic viruses can explain the variation of carbon export efficiency

**Figure 2:**
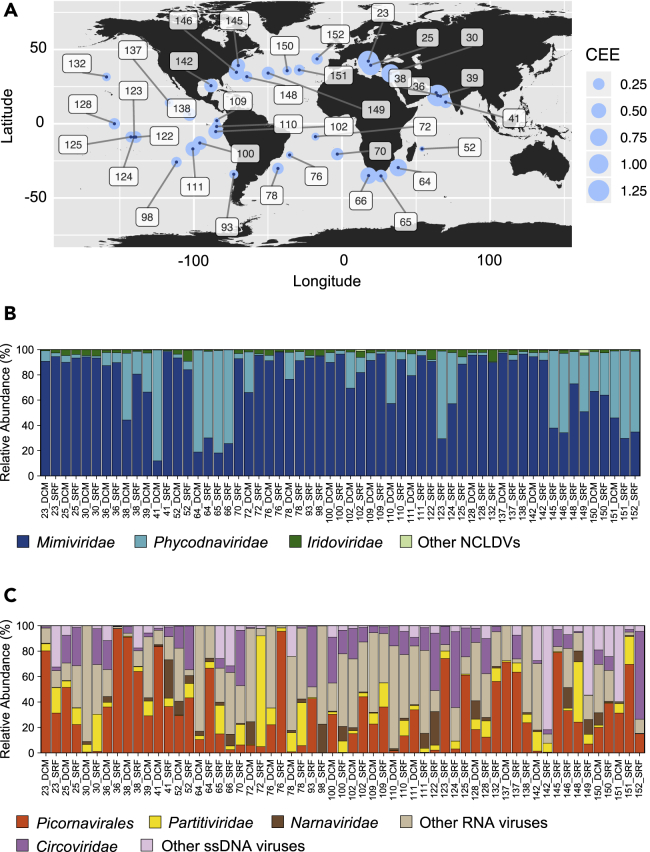
Carbon export efficiency and relative marker-gene occurrence of eukaryotic plankton viruses along the sampling route (A) Carbon export efficiency (CEE) estimated at 39 *Tara* Oceans stations where surface and DCM layers were sampled for prokaryote-enriched metagenomes and eukaryotic metatranscriptomes. See also Figures S5 and S11. (B and C) Relative marker-gene occurrence of major groups of viruses of eukaryotic plankton for NCLDVs in metagenomes (B) and for RNA and ssDNA viruses in metatranscriptomes (C) at 59 sampling sites.

**Figure 3:**
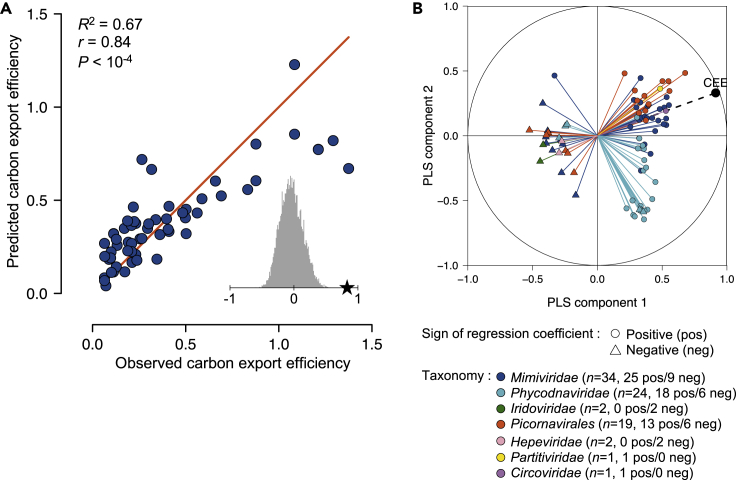
Relative abundance of eukaryotic plankton viruses is associated with carbon export efficiency in the global ocean (A) Bivariate plot between predicted and observed values in a leave-one-out cross-validation test for carbon export efficiency. The PLS regression model was constructed using occurrence profiles of 1,523 marker-gene sequences (1,309 PolBs, 180 RdRPs, and 34 Reps) derived from environmental samples. *r*, Pearson correlation coefficient; *R*^2^, the coefficient of determination between measured response values and predicted response values. *R*^2^, which was calculated as 1—SSE/SST (sum of squares due to error and total) measures how successful the fit is in explaining the variance of the response values. The significance of the association was assessed using a permutation test (*n* = 10,000) (gray histogram in (A)). The red diagonal line shows the theoretical curve for perfect prediction. (B) Pearson correlation coefficients between CEE and occurrence profiles of 83 viruses that have VIP scores >2 (VIPs) with the first two components in the PLS regression model using all samples. PLS components 1 and 2 explained 83% and 11% of the variance of CEE, respectively. Fifty-eight VIPs had positive regression coefficients in the model (shown with circles), and 25 had negative regression coefficients (shown with triangles). See also Figures S6, S7, and S12, Table S1, and Data S1.

Among the PolB, RdRP, and Rep sequences identified in the *Tara* Oceans gene catalogs, 38%, 18%, and 11% (total = 1,523 sequences), respectively, were present in at least five samples and had matching carbon export measurement data (Table 1). We used the relative abundance (defined as the centered log-ratio transformed gene-length normalized read count) profiles of these 1,523 marker-gene sequences at 59 sampling sites in the photic zone of 39 *Tara* Oceans stations (Figure 2) to test for association between their composition and a measure of carbon export efficiency (CEE, see Transparent Methods, Figure S5). A partial least squares (PLS) regression model explained 67% (coefficient of determination *R*^2^ = 67%) of the variation in CEE with a Pearson correlation coefficient of 0.84 between observed and predicted values. This correlation was confirmed to be statistically significant by permutation test (p < 1 × 10^−4^) (Figure 3A).

We also tested for their association with estimates of carbon export flux at 150 meters (CE_150_) and NPP. PLS regressions explained 54% and 64% of the variation in CE_150_ and NPP with Pearson correlation coefficients between observed and predicted values of 0.74 (permutation test, p < 1 × 10^−4^) and 0.80 (permutation test, p < 1 × 10^−4^), respectively (Figure S6). In these three PLS regression models, 83, 86, and 97 viruses were considered to be key predictors (i.e., Variable Importance in the Projection [VIP] score > 2) of CEE, CE_150_, and NPP, respectively. PLS models for NPP and CE_150_ shared a larger number of predictors (52 viruses) compared with the PLS models for NPP and CEE (seven viruses) (two-proportion Z-test, p = 4.14 × 10^−12^). Consistent with this observation, CE_150_ was correlated with NPP (Pearson’s *r* = 0.77; parametric test, p < 1 × 10^−12^). This result implies that the magnitude of export in the analyzed samples was partly constrained by primary productivity. However, CEE was not correlated with NPP (*r* = 0.16; parametric test, p = 0.2) or CE_150_ (*r* = 0.002; parametric test, p = 0.99). Thus, as expected, primary productivity was not a major driver for the efficiency of carbon export.

To assess the sensitivity of the model to the definition of carbon export efficiency, we employed an alternative measure of carbon export efficiency that considers euphotic zone depth (T_100_, see Transparent Methods). T_100_ was correlated with CEE (*r* = 0.66; parametric test, p < 1 × 10^−8^), and PLS regression explained 44% of the variation in T_100_ (permutation test, p < 1 × 10^−4^) (Figure S7). Of 72 predictors of the PLS model for T_100_, 30 were shared with that for CEE. This result demonstrates the robustness of the PLS model to definitions of carbon export efficiency.

**Figure 4:**
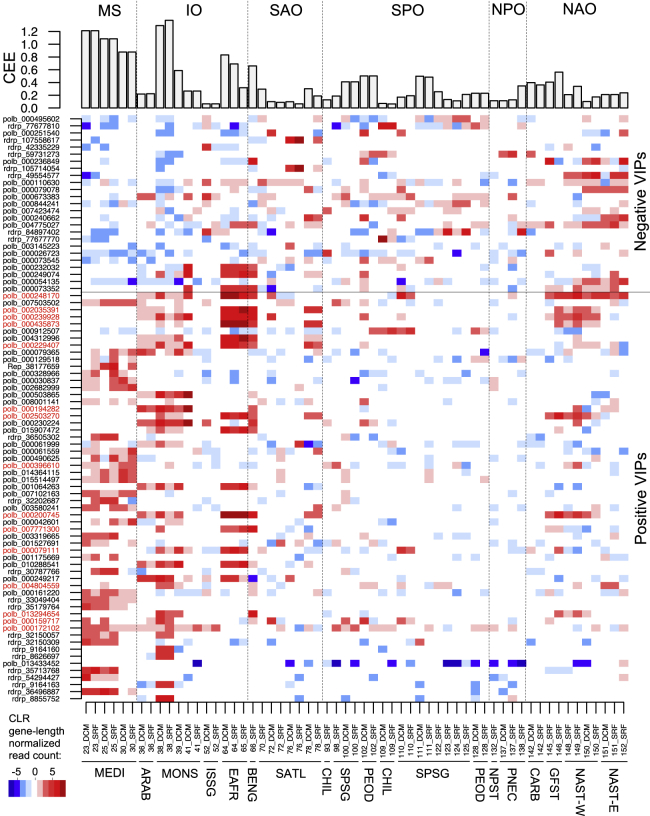
Biogeography of viruses associated with carbon export efficiency The upper panel shows carbon export efficiency (CEE = CE_deep_/CE_surface_) for 59 sampling sites. The bottom panel is a map reflecting relative abundances, expressed as centered log-ratio transformed, gene-length normalized read counts of viruses positively and negatively associated with CEE that have VIP scores >2 (VIPs). MS, Mediterranean Sea; IO, Indian Ocean; SAO, South Atlantic Ocean; SPO, South Pacific Ocean; NPO, North Pacific Ocean; NAO, North Atlantic Ocean. The bottom horizontal axis is labeled with *Tara* Oceans station numbers, sampling depth (SRF, surface; DCM, deep chlorophyll maximum), and abbreviations of biogeographic provinces. Viruses labeled in red correspond to positive VIPs that are highly represented in one or more biogeographic provinces outside MS and IO.

The 83 viruses (5% of the viruses included in our analysis) that were associated with CEE with a VIP score > 2 are considered to be important predictors of CEE in the PLS regression (Figure 3B, Data S1), and these viruses are hereafter referred to as VIPs (Viruses Important in the Prediction). Fifty-eight VIPs had positive regression coefficient, and 25 had negative regression coefficient in the prediction (Figure 3B). Most of the positively associated VIPs showed high relative abundance in the Mediterranean Sea and in the Indian Ocean where CEE tends to be high compared with other oceanic regions (Figure 4). Among them, 15 (red labels in Figure 4) also had high relative abundance in samples from other oceanic regions, showing that these viruses are associated with CEE at a global scale. In contrast, negatively associated VIPs tend to have higher relative abundance in the Atlantic Ocean and the Southern Pacific Ocean where CEE is comparatively lower. In the following sections, we investigate potential hosts of the VIPs in order to interpret the statistical association between viral community composition and CEE in the light of previous observations in the literature.

### Viruses correlated with CEE infect ecologically important hosts

Most of the VIPs (77 of 83) belong to *Mimiviridae* (*n* = 34 with 25 positive VIPs and 9 negative VIPs), *Phycodnaviridae* (*n* = 24 with 18 positive VIPs and 6 negative VIPs), and ssRNA viruses of the order *Picornavirales* (*n* = 19 with 13 positive VIPs and 6 negative VIPs) (Figure 3B, Table S1). All the phycodnavirus VIPs were most closely related to prasinoviruses infecting Mamiellales, with amino acid sequence percent identities to reference sequences ranging between 35% and 95%. The six remaining VIPs were two NCLDVs of the family *Iridoviridae* negatively associated with CEE, three RNA viruses (two ssRNA viruses of the family *Hepeviridae* negatively associated with CEE and one dsRNA virus of the family *Partitiviridae* positively associated with CEE), and one ssDNA virus of the family *Circoviridae* positively associated with CEE. A proportionally larger number of PolBs were included in the model than RdRP and Rep sequences depending on their representations in the input data. Therefore, the larger number of NCLDV VIPs obtained does not necessarily mean that this group of viruses is more important than others regarding their association with CEE.

Host information may help understand the relationship between these VIPs and CEE. We performed genomic context analysis for PolB VIPs and phylogeny-guided network-based host prediction for PolB and RdRP to infer putative relationship between virus and host (see Transparent Methods).

Taxonomic analysis of genes predicted in 10 metagenome-assembled genomes (MAGs) from the eukaryotic size fractions and 65 genome fragments (contigs) assembled from the prokaryotic size fraction encoding VIP PolBs further confirmed their identity as *Mimiviridae* or *Phycodnaviridae* (Figure S8). The size of MAGs ranged between 30 kbp and 440 kbp with an average of 210 kbp (Table S2). The presence of genes with high-sequence similarities to cellular genes in a viral genome is suggestive of a relationship between virus and host (Monier et al., 2009; Yoshikawa et al., 2019). Two closely related *Mimiviridae* VIPs, PolB 000079111 (positively associated with CEE) and PolB 000079078 (negatively associated with CEE), were phylogenetically close to the pelagophyte virus *Aureococcus anophagefferens virus* (AaV). One MAG (268 kbp in size) corresponding to PolB 000079111 encoded seven genes showing high similarities to genes from Pelagophyceae, and another MAG (382 kbp in size), corresponding to PolB 000079078, encoded five genes similar to genes from Pelagophyceae. All but one of these 12 genes were encoded on a genome fragment containing genes annotated as viral, including five NCLDV core genes (Data S2), excluding the possibility of contamination in these MAGs. Two closely related *Phycodnaviridae* VIPs, PolB 001064263 and 010288541, were positively associated with CEE. Both of these PolBs correspond to an MAG (134 kbp in size) encoding one gene likely derived from Mamiellales. The genomic fragment harboring this cellular gene was found to encode 10 genes annotated as viral (Data S2).

We conducted a phylogeny-guided, network-based host prediction analysis for *Mimiviridae*, *Phycodnaviridae*, and *Picornavirales* (Figures S9 and S10). Only a subset of the VIPs was included in this analysis because we kept the most reliable sequences (n = 44) to obtain a well-resolved tree topology. Within the *Prasinovirus* clade, which contained thirteen VIPs (nine positive and four negative), seven different eukaryotic orders were detected as predicted host groups for ten nodes in the tree. Mamiellales*,* the only known host group of prasinoviruses, was detected at eight nodes (five of them had no parent-to-child relationships), whereas the other six eukaryotic orders were found at only one node (or two in the case of Eutreptiales) (Figure S9). The order Mamiellales includes three genera (*Micromonas*, *Ostreococcus*, and *Bathycoccus*), which are bacterial-sized green microalgae common in coastal and oceanic environments and are considered to be influential actors in oceanic systems (Monier et al., 2016). Various prasinoviruses (fourteen with available genome sequences) have been isolated from the three genera.

Within the family *Mimiviridae*, which contains fifteen VIPs (ten positive and five negative), twelve different orders were predicted as putative host groups (Figure S9). Collodaria was detected at fifteen nodes (two of them had no parent-to-child relationships), and Prymnesiales at six nodes (three of them had no parent-to-child relationships), whereas all other orders were present at a maximum of one node each with no parent-to-child relationships. The nodes enriched for Prymnesiales and Collodaria fell within a monophyletic clade (marked by a red arrow in Figure S9) containing four reference haptophyte viruses infecting Prymnesiales and two reference haptophyte viruses infecting Phaeocystales. Therefore, the environmental PolB sequences in this *Mimiviridae* clade (including five positive VIPs and one negative VIP) are predicted to infect Prymnesiales or related haptophytes. The detection of Collodaria may be the result of indirect associations that reflect a symbiotic relationship with Prymnesiales, as some acantharians, evolutionarily related to the Collodaria, are known to host Prymnesiales species (Mars Brisbin et al., 2018). Known species of Prymnesiales and Phaeocystales have organic scales, except one Prymnesiales species, *Prymnesium neolepis*, which bears siliceous scales (Yoshida et al., 2006). Previous studies revealed the existence of diverse and abundant noncalcifying picohaptophytes in open oceans (Endo et al., 2018; Liu et al., 2009). Clear host prediction was not made for the other nine *Mimiviridae* VIPs shown in the phylogenetic tree. Three VIPs (two positive and one negative) in the tree were relatives of AaV. One negatively associated VIP was a relative of *Cafeteria roenbergensis virus* infecting a heterotrophic protist. The five remaining *Mimiviridae* VIPs are very distant from any known *Mimiviridae*.

Sixteen *Picornavirales* VIPs (eleven positive and five negative) were included in the phylogeny-guided, network-based host prediction analysis (Figure S10). Nine (seven positive and two negative) were grouped within *Dicistroviridae* (known to infect insects) and may therefore infect marine arthropods such as copepods, the most ubiquitous and abundant mesozooplankton groups involved in carbon export (Turner, 2015). Three other *Picornavirales* VIPs were placed within a clade containing known bacillarnaviruses. Two of them (35179764 and 33049404) were positively associated with CEE and had diatoms of the order Chaetocerotales as a predicted host group. The third one (107558617) was negatively associated with CEE and distant from other bacillarnaviruses and had no host prediction. Diatoms have been globally observed in the deep sea (Agusti et al., 2015; Leblanc et al., 2018) and identified as important contributors of the biological carbon pump (Tréguer et al., 2018). One positively associated VIP (32150309) was in a clade containing *Aurantiochytrium single-stranded RNA virus* (AsRNAV), infecting a marine fungoid protist thought to be an important decomposer (Takao et al., 2005). The last three *Picornavirales* VIPs (59731273, 49554577, and 36496887) had no predicted host and were too distant from known *Picornavirales* to speculate about their putative host group.

Outside *Picornavirales*, three RNA virus VIPs (two *Hepeviridae*, negatively associated, and one *Partitiviridae*, positively associated) were identified, for which no reliable host inferences were made by sequence similarity. Known *Hepeviridae* infect metazoans, and known *Partitiviridae* infect fungi and plants. The two *Hepeviridae*-like viruses were most closely related to viruses identified in the transcriptomes of mollusks (amino acid identities of 48% for 42335229 and 43% for 77677770) (Shi et al., 2016). The *Partitiviridae*-like VIP (35713768) was most closely related to a fungal virus, *Penicillium stoloniferum virus S* (49% amino acid identity)*.*

One ssDNA virus VIP (38177659) was positively associated with CEE. It was annotated as a *Circoviridae*, although it groups with other environmental sequences as an outgroup of known *Circoviridae*. This VIP was connected with copepod, mollusk, and Collodaria OTUs in the co-occurrence network but no enrichment of predicted host groups was detected for its clade. *Circoviridae*-like viruses are known to infect copepods (Dunlap et al., 2013) and have been reported to associate with mollusks (Dayaram et al., 2015), but none have been reported for Collodaria.

Overall, we could infer hosts for 37 VIPs (Tables 2 and S3). Most of the predicted hosts are known to be ecologically important as primary producers (Mamiellales, Prymnesiales, Pelagophyceae, and diatoms) or grazers (copepods). Of these, diatoms and copepods are well known as important contributors to the BCP but others (i.e., Mamiellales, Prymnesiales, Pelagophyceae) have not been recognized as major contributors to the BCP. Our analysis also revealed that positive and negative VIPs are not separated in either the viral or host phylogenies.

**Table 2:** Host predictions per viral and host group for viruses associated with carbon export efficiency

Virus-Host Relationship	Positive VIPs^a^	Negative VIPs^a^	Total
NCLDV-mamiellales	10	4	15
NCLDV-prymnesiales	5	1	6
NCLDV-pelagophyceae	2	1	3
NCLDV-no prediction	26	11	36
RNA virus-copepoda	7	2	9
RNA virus-chaetocerotales	2	0	2
RNA virus-labyrinthulomycetes	1	0	1
RNA virus-no prediction	4	6	10
ssDNA virus-copepoda	1	0	1
Total	58	25	83

See also Figures S8–S10, Tables S2 and S3, and Data S2.

a VIPs refers to viruses having VIP scores > 2. Positive and negative VIPs had positive and negative regression coefficients in the PLS model, respectively.

### Viruses positively correlated with CEE tend to interact with silicified organisms

The phylogeny-guided, network-based host prediction analysis correctly predicted known relationships between virus and host (for viruses infecting Mamiellales, Prymnesiales, and Chaetocerotales) using our large dataset, despite the reported limitations of these co-occurrence network-based approaches (Coenen and Weitz, 2018). This result prompted us to further exploit the species co-occurrence networks (Table S4) to investigate functional differences between the eukaryotic organisms predicted to interact with positive VIPs, negative VIPs, and viruses less important for prediction of CEE (VIP score <2) (non-VIPs). For this purpose, we used literature-based functional trait annotations associated with eukaryotic meta-barcodes (see Transparent Methods). Positive VIPs had a greater proportion of connections with silicified eukaryotes (*Q* = 0.001) but not with chloroplast-bearing eukaryotes (*Q* = 0.16) nor calcifying eukaryotes (*Q* = 1), compared to non-VIPs (Table 3). No functional differences were observed between negative VIPs and non-VIPs viruses (Table S5) or positive VIPs (Table S6).

**Table 3:** Functional differences between eukaryotes found to be best connected to positively associated and not associated with carbon export efficiency

Functional trait	Positive VIPs^a^ (*n* = 50)		Non-VIPs^a^ (*n* = 983)		p value (Fisher's exact test, two sided)	Adjusted p value (BH) (*Q*)
	Presence	Absence	Presence	Absence		
Chloroplast	20	30	276	690	0.109	0.164
Silicification	11	39	60	920	0.000	0.001
Calcification	1	49	30	950	1.000	1.000

See also Tables S4–S6.

a VIPs refer to viruses having VIP scores > 2. Positive VIPs had positive regression coefficients in the PLS model.

### Multifarious ways viruses affect the fate of carbon

Our analysis revealed that eukaryotic virus composition was able to predict CEE in the global sunlit ocean, and 83 out of the 1,523 viruses had a high importance in the predictive model. This association is not a proof that the viruses are the cause of the variation of CEE. Viruses, especially those showing latent/persistent infections (Goic and Saleh, 2012), may be found to be associated with CEE if their host affects CEE regardless of viral infection. Organisms that preferentially grow in marine snow (Bochdansky et al., 2017) may also bring associations between viruses infecting those organisms and CEE. Alternatively, the observed associations between VIPs and CEE may reflect a more direct causal relationship, which we attempt to explore in light of the large body of literature on the mechanisms by which viruses impact the fate of carbon in the oceans.

Among the 83 VIPs, 58 were positively associated with CEE. Such a positive association is expected from the “viral shuttle” model, which states that viral activity could facilitate carbon export to the deep ocean (Fuhrman, 1999; Sullivan et al., 2017; Weinbauer, 2004), because a virus may induce secretion of sticky material that contributes to cell/particle aggregation, such as transparent exopolymeric particles (TEP) (Nissimov et al., 2018). We found that CEE (i.e., CE_deep_/CE_surface_) increased with the change of particles size from surface to deep (*ρ* = 0.42, p = 8 × 10^−9^) (Figure S11). This positive correlation may reflect an elevated level of aggregation in places where CEE is high, although it could be also due to the presence of large organisms at depth.

Greater aggregate sinking along with higher particulate carbon fluxes was observed in North Atlantic blooms of *Emiliania huxleyi* that were infected early by the virus EhV, compared with late-infected blooms (Laber et al., 2018). In the same bloom, viral infection stage was found to proceed with water column depth (Sheyn et al., 2018). No EhV-like PolB sequences were detected in our dataset, which was probably due to sampled areas and seasons.

Laboratory experiments suggest that viruses closely related to positive VIPs, such as prasinoviruses, have infectious properties that may drive carbon export. Cultures of *Micromonas pusilla* infected with prasinoviruses showed increased TEP production compared with non-infected cultures (Lønborg et al., 2013). The hosts of prasinoviruses (Mamiellales) have been proposed to contribute to carbon export in the western subtropical North Pacific (Shiozaki et al., 2019). Some prasinoviruses encode glycosyltransferases (GTs) of the GT2 family. The expression of GT2 family members during infection possibly leads to the production of a dense fibrous hyaluronan network and may trigger the aggregation of host cells (Van Etten et al., 2017) with an increase in the cell wall C:N ratio. We detected one GT2 in an MAG of two *Phycodnaviridae*-like positive VIPs (000200745 and 002503270) predicted to infect Mamiellales, one in an MAG corresponding to the putative pelagophyte positive VIP 000079111 related to AaV and six in two MAGs (three each) corresponding to two *Mimiviridae*-like positive VIPs (000328966 and 001175669). *Phaeocystis globosa virus* (PgV), closely related to the positive VIP PolB 000912507 (Figure S9), has been linked with increased TEP production and aggregate formation during the termination of a *Phaeocystis* bloom (Brussaard et al., 2007). Two closely related bacillarnavirus VIPs were positively associated with CEE and predicted to infect Chaetocerales. A previous study revealed an increase in abundance of viruses infecting diatoms of *Chaetoceros* in both the water columns and the sediments during the bloom of their hosts in a coastal area (Tomaru et al., 2011), suggesting sinking of cells caused by viruses. Furthermore, the diatom *Chaetoceros tenuissimus* infected with a DNA virus (CtenDNAV type II) has been shown to produce higher levels of large-sized particles (50–400 μm) compared with non-infected cultures (Tomaru et al., 2011; Yamada et al., 2018).

The other 25 VIPs were negatively associated with CEE. This association is compatible with the “viral shunt,” which increases the amount of DOC (Wilhelm and Suttle, 1999) and reduces the transfer of carbon to higher trophic levels and to the deep ocean (Fuhrman, 1999; Weitz et al., 2015). Increased DOC has been observed in culture of Mamiellales lysed by prasinoviruses (Lønborg et al., 2013). A field study reported that PgV, to which the negative VIP PolB 000054135 is closely related (Figure S9), can be responsible for up to 35% of cell lysis per day during bloom of its host (Baudoux et al., 2006), which is likely accompanied by consequent DOC release. Similarly, the decline of a bloom of the pelagophyte *Aureococcus anophagefferens* has been associated with active infection by AaV (to which one negative VIP is closely related) (Moniruzzaman et al., 2017). Among RNA viruses, eight were negative VIPs (six *Picornavirales* and two *Hepeviridae*). The higher representation of *Picornavirales* in the virioplankton (Culley, 2018) than within cells (Urayama et al., 2018) suggests that they are predominantly lytic, although no information exists regarding the effect of *Picornavirales* on DOC release.

It is likely that the “viral shunt” and “shuttle” simultaneously affect and modulate CEE in the global ocean (Zimmerman et al., 2019). The relative importance of these two phenomena must fluctuate considerably depending on the host traits, viral effects on metabolism, stages of infection, and environmental conditions. Reflecting this complexity, viruses of a same host group could be found to be either positively or negatively associated with CEE. We found that even two very closely related *Mimiviridae* viruses (PolBs 000079111 and 000079078 sharing 94% nucleotide identity over their full gene lengths) most likely infecting pelagophyte algae were positively and negatively associated with CEE.

Five percent of the tested viruses were associated with CEE in our study. Similarly, 4% and 2% of bacterial virus populations were found to be associated with the magnitude of carbon export (Guidi et al., 2016) and CEE (Figure S12), respectively. These results suggest that viruses affecting CEE are rather uncommon. It is plausible that such viruses affect CEE by infecting organisms that are functionally important (abundant or keystone species), as we observed in host prediction. The vast majority (95%) of non-VIPs may not have a significant impact on CEE, because they do not strongly impact the host population, for instance, by stably coexisting with their hosts. It is worth noting that experimental studies have reported cultures of algae with viruses that reach a stable co-existence state after a few generations (Yau et al., 2020).

### Conclusions

Eukaryotic virus community composition was able to predict CEE at 59 sampling sites in the photic zone of the world ocean. This statistical association was detected based on a large omics dataset collected throughout the oceans and processed with standardized protocols. The predictability of CEE by viral composition is consistent with the hypothesis that “viral shunt” and “shuttle” are functioning at a global scale. Among 83 viruses with a high importance in the prediction of CEE, 58 viruses were positively and 25 negatively correlated with carbon export efficiency. Most of these viruses belong to *Prasinovirus*, *Mimiviridae*, and *Picornavirales* and are either new to science or with no known roles in carbon export efficiency. Thirty-six of these “select” viruses were predicted to infect ecologically important hosts such as green algae of the order Mamiellales, haptophytes, diatoms, and copepods. Positively associated viruses had more predicted interactions with silicified eukaryotes than non-associated viruses did. Overall, these results imply that the effect of viruses on the “shunt” and “shuttle” processes could be dependent on viral hosts and ecosystem dynamics.

### Limitations of the study

The observed statistical associations between viral compositions and examined parameters (i.e., CEE, CE and NPP) do not convey the information about the direction of their potential causality relationships, and they could even result from indirect relationships as discussed earlier. Certain groups of viruses detected in samples may be over- or underrepresented because of the technical limitations in size fractionation, DNA/RNA extraction, and sequencing.

### Resource availability

#### Lead contact

Further information and requests for resources should be directed to and will be fulfilled by Lead Contact, Hiroyuki Ogata (ogata@kuicr.kyoto-u.ac.jp).

#### Materials availability

This study did not generate unique reagent.

#### Data and code availability

The authors declare that the data supporting the findings of this study are available within the paper and its supplemental files, as well as at the GenomeNet FTP: ftp://ftp.genome.jp/pub/db/community/tara/Cpump/Supplementary_material/.

Our custom R script used to test for association between viruses and environmental variables (CEE, CE_150_, NPP and T_100_) is available along with input data at the GenomeNet FTP: ftp://ftp.genome.jp/pub/db/community/tara/Cpump/Supplementary_material/PLSreg/. The Taxon Interaction Mapper (TIM) tool developed for this study and used for virus host prediction is available at https://github.com/RomainBlancMathieu/TIM.

## Methods

All methods can be found in the accompanying Transparent Methods supplemental file.

## Supplemental information

Document S1. Transparent methods, Figures S1–S12, and Tables S1–S6
