# Supplementary material for: Eukaryotic virus composition can predict the efficiency of carbon export in the global ocean
Source: iScience. 2020 Dec 29;24(1):102002. doi: 10.1016/j.isci.2020.102002 (PMC7811142; doi:10.1016/j.isci.2020.102002)
Supplement: Document S1. Transparent methods, Figures S1–S12, and Tables S1–S6 [file mmc1.pdf]

iScience, Volume *24*

Supplemental Information

Eukaryotic virus composition can predict the efficiency of carbon export in the global ocean

Hiroto Kaneko, Romain Blanc-Mathieu, Hisashi Endo, Samuel Chaffron, Tom O. Delmont, Morgan Gaia, Nicolas Henry, Rodrigo Hernández-Velázquez, Canh Hao Nguyen, Hiroshi Mamitsuka, Patrick Forterre, Olivier Jaillon, Colomban de Vargas, Matthew B. Sullivan, Curtis A. Suttle, Lionel Guidi, and Hiroyuki Ogata

Supplemental Figures

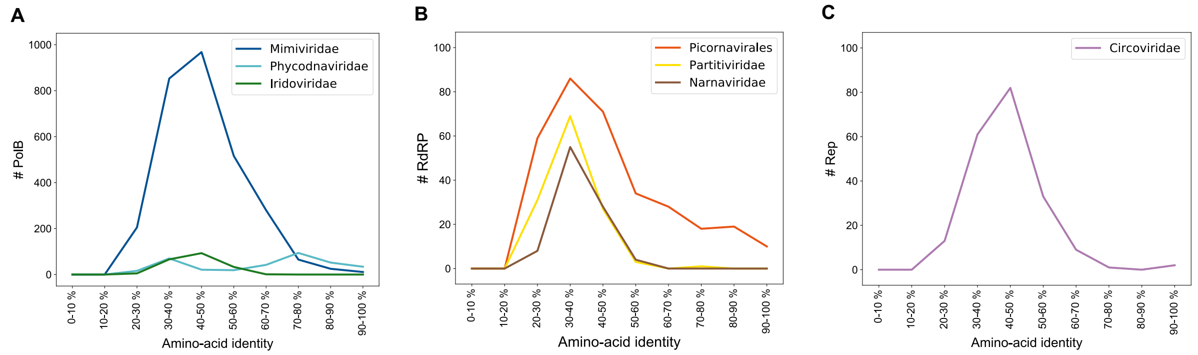

Figure S1. Distribution of the degree of amino acid identity between environmental sequences and their best BLAST hits to reference sequences, Related to Figure 1. (A) Nucleocytoplasmic large DNA viruses (NCLDVs). (B) RNA viruses. (C) ssDNA viruses.

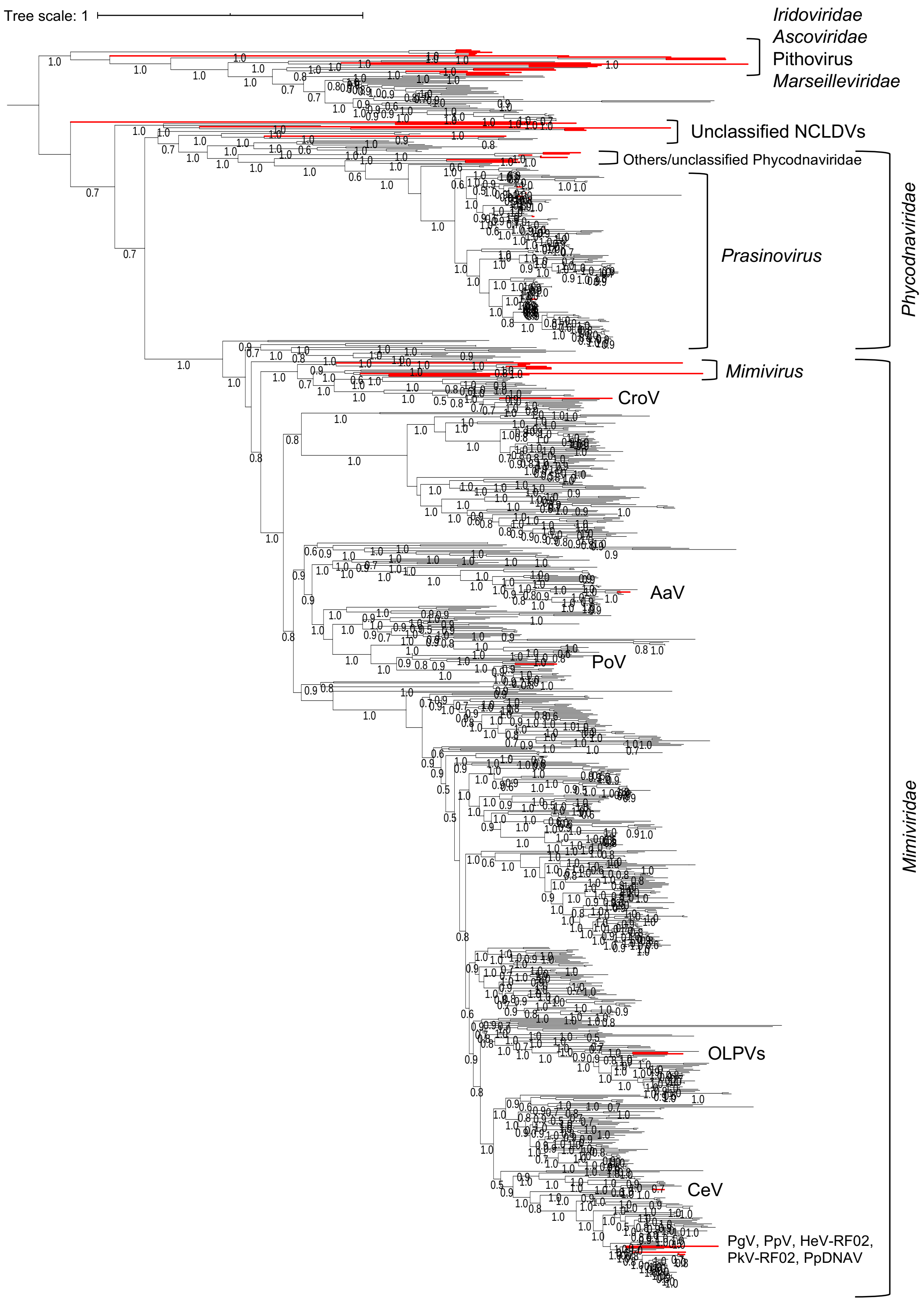

Figure S2. Maximum likelihood phylogenetic trees for NCLDV DNA polymerase family B, Related to Figure 1A. Environmental sequences are shown in black and references in red. Approximate Shimodaira–Hasegawa (SH)-like local support values greater than 0.8 are shown. Scale bar indicates one change per site.

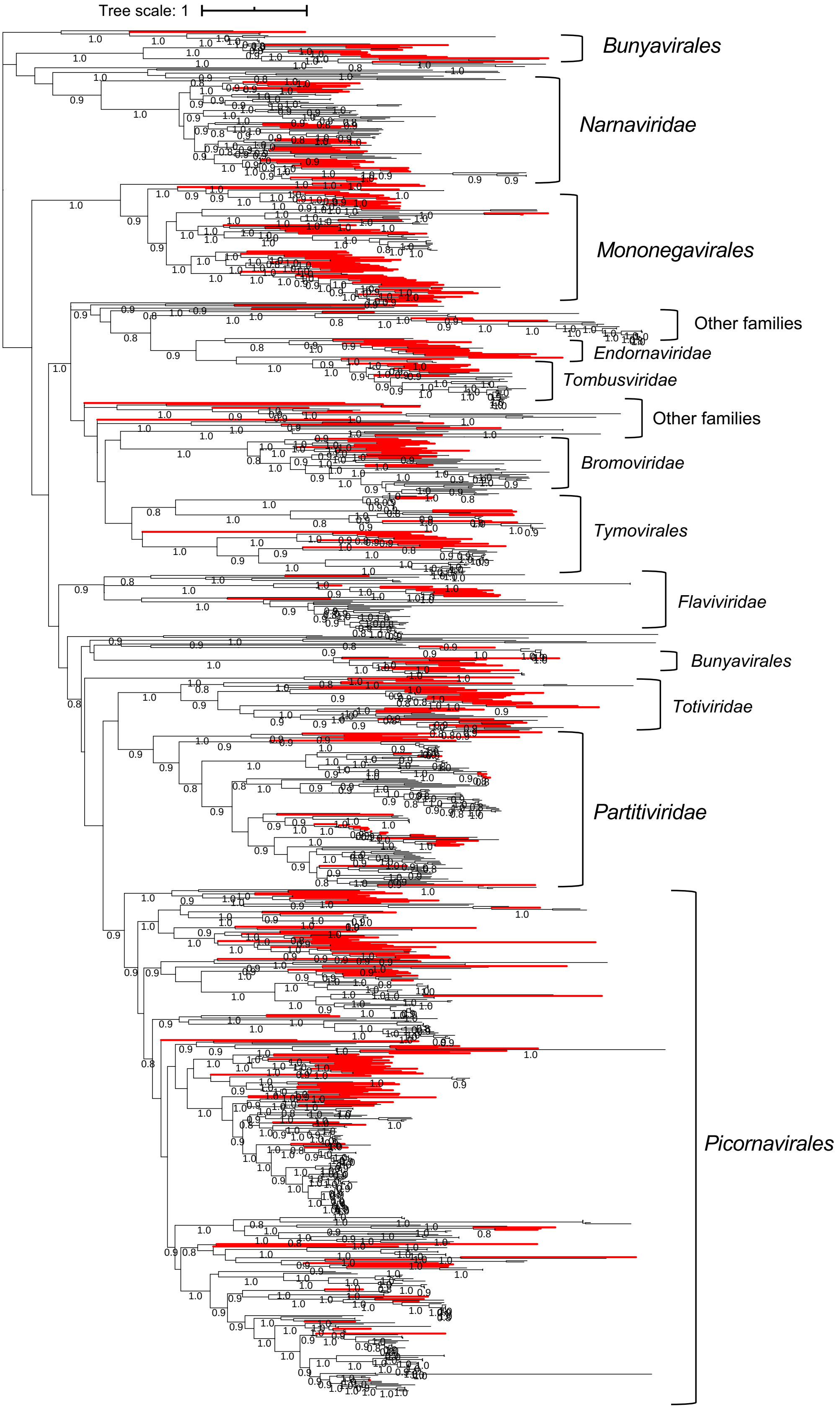

Figure S3. Unrooted maximum likelihood phylogenetic trees for RNA virus RNA-dependent RNA polymerase, Related to Figure 1B. Environmental sequences are shown in black and references in red. Approximate SH-like local support values greater than 0.8 are shown. Scale bar indicates one change per site.

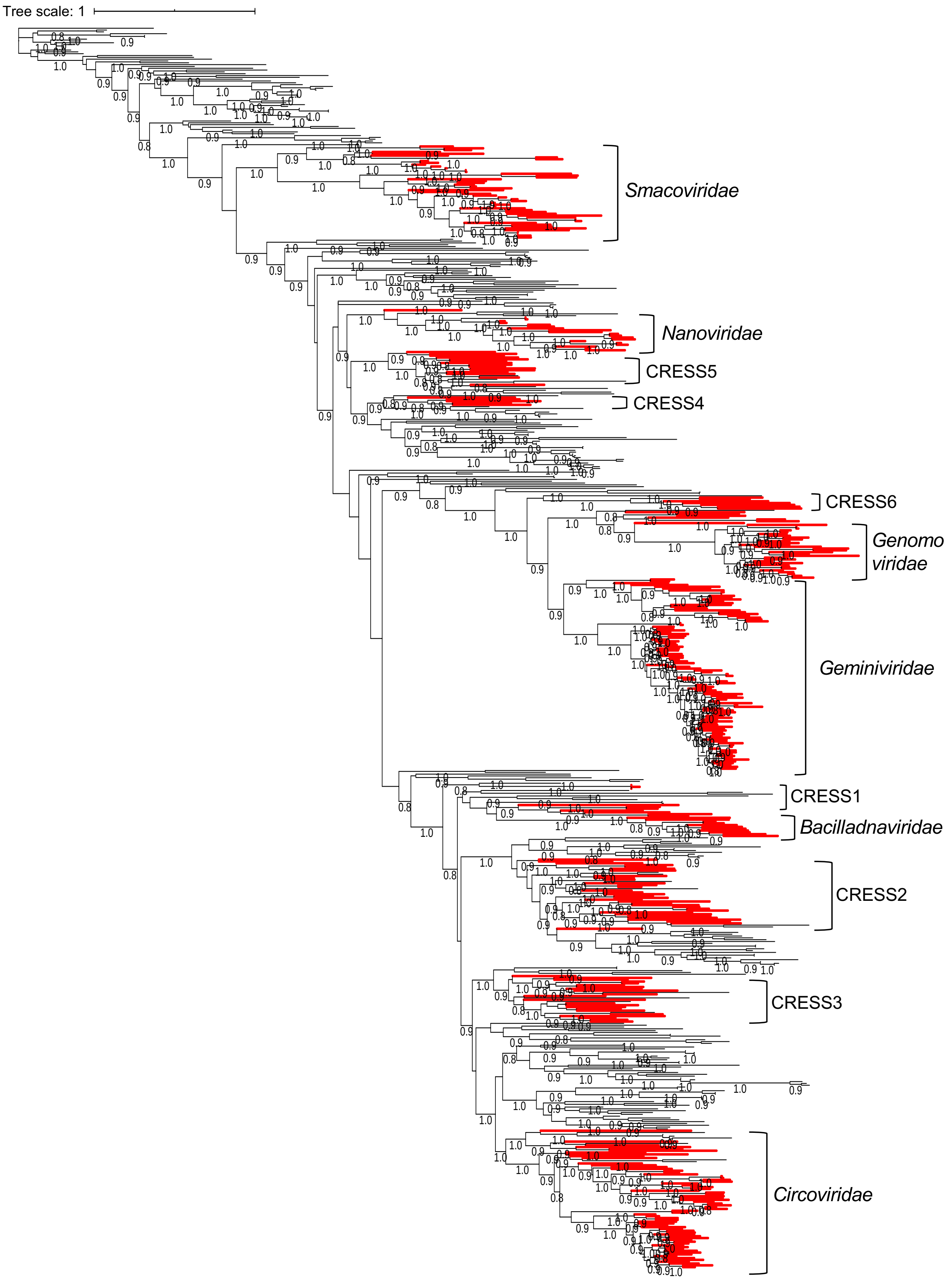

Figure S4: Unrooted maximum likelihood phylogenetic trees for ssDNA virus replication-associated protein, Related to Figure 1C. Environmental sequences are shown in black and references in red. Approximate SH-like local support values greater than 0.8 are shown. Scale bar indicates one change per site.

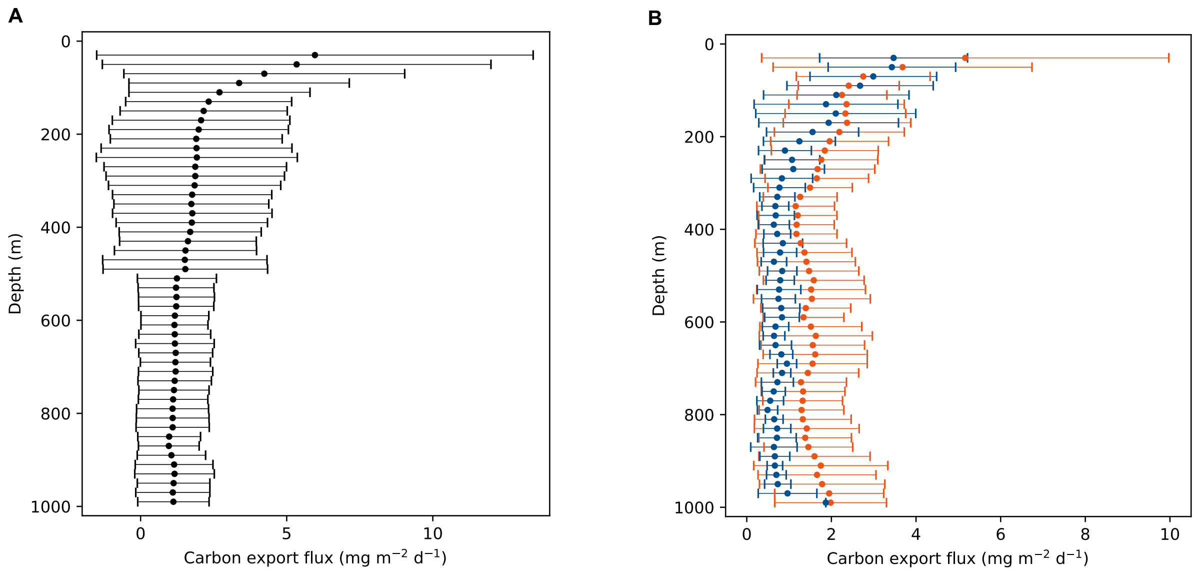

Figure S5. Variation in carbon export flux (mg m^{-2} d^{-1}) across sampling depths in the water column, Related to Figure 2A; Transparent Methods. Dots are average values, and horizontal lines represent standard deviation. (A) All sampling sites. (B) Red shows the carbon flux profile of Indian Monsoon Gyres (MONS) where mean CEE is relatively high (0.41) and blue shows that of North Atlantic Subtropical Gyres (West) (NAST-W) where mean CEE is relatively low (0.26).

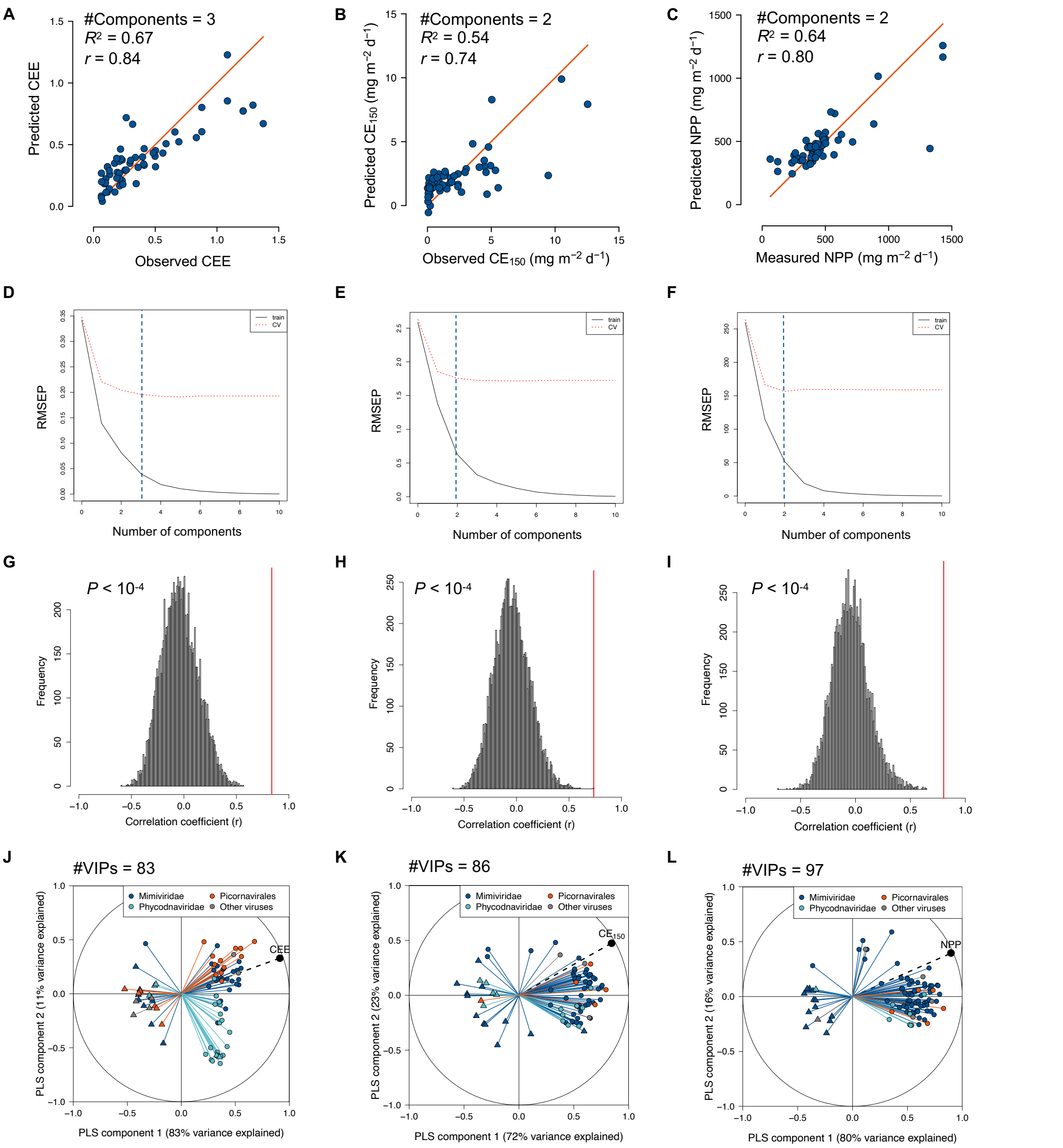

Figure S6. The results of PLS regressions using relative abundance profiles of viral marker-genes to explain the variance of CEE, CE_{150} and NPP, Related to Figure 3. (A-C) Bivariate plots between predicted and observed response values in a leave-one-out cross-validation test. (A) for CEE, (B) for CE_{150} and (C) for NPP. The red diagonal line shows the theoretical curve for perfect prediction. (D-F) Variation in root mean squared error of predictions (RMSEP) for the training set (solid black line) and cross-validation set (red dashed line) across the number of components. (D) for CEE, (E) for CE_{150} and (F) for NPP. Blue dashed line shows the number of components selected for the analysis. (G-I) Results of the permutation tests (n = 10,000) supporting the significance of the association between viruses and the response variable. (G) for CEE, (H) for CE_{150} and (I) for NPP. The histograms show the distribution of Pearson correlation coefficients obtained from PLS models reconstructed based on the permutated response variable and red line show the non-permutated response variable. (J-L) Pearson correlation coefficients between the response variable and abundance profiles of viruses with VIP scores > 2 (VIPs) with the first two components in the PLS regression model using all samples. (J) for CEE, (K) for CE_{150} and (L) for NPP. Viruses with positive regression coefficients are shown with circles, and those with negative coefficients are shown with triangles.

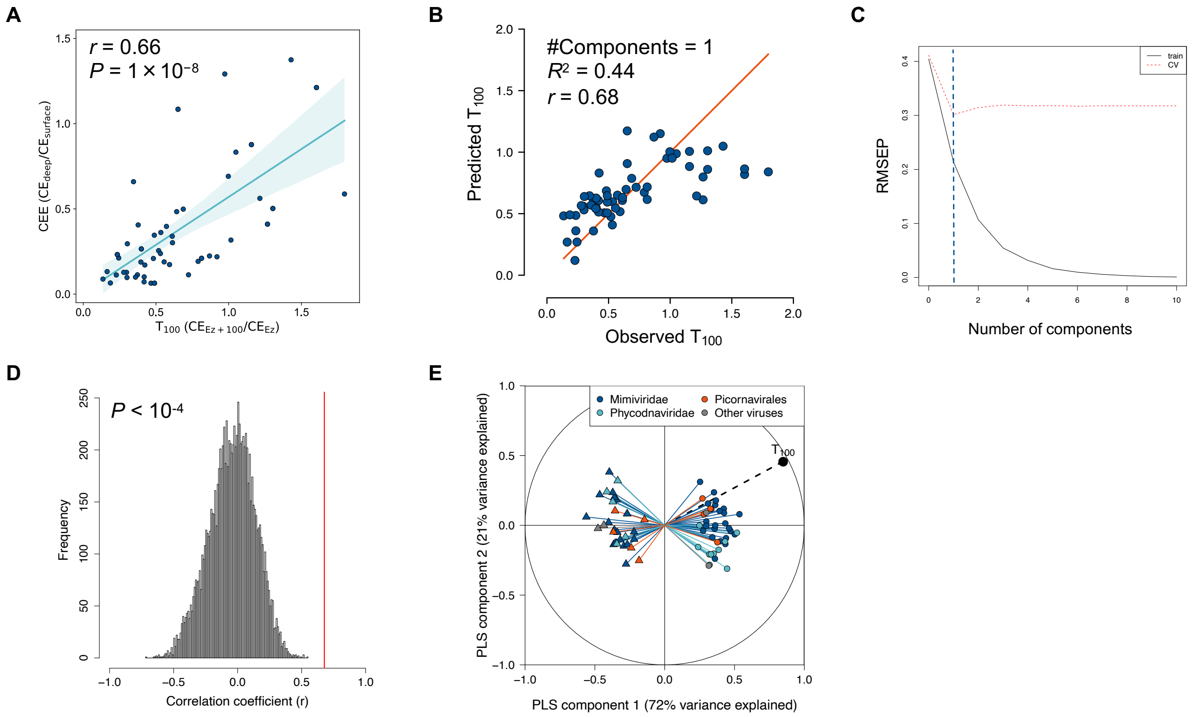

Figure S7. The assessment of the sensitivity of the model to the definition of carbon export efficiency, Related to Figure 3. (A) CEE defined as $CE_{deep}/CE_{surface}$ is well correlated with alternative index of carbon export efficiency defined as CE_{Ez+100}/CE_{Ez} (T_{100}). (B-E) The result of PLS regression using relative abundance profiles of viral marker-genes to explain T_{100}. (B) Bivariate plots between predicted and observed response values in a leave-one-out cross-validation test. (C) Variation in root mean squared error of predictions (RMSEP) across the number of components. (D) Results of the permutation tests (n = 10,000) supporting the significance of the association between viruses and the response variable. (E) Pearson correlation coefficients between the response variable and abundance profiles of viruses with VIP scores > 2 (VIPs) with the first two components in the PLS regression model using all samples. See the legend of Figure S6 for detailed explanation of figures.

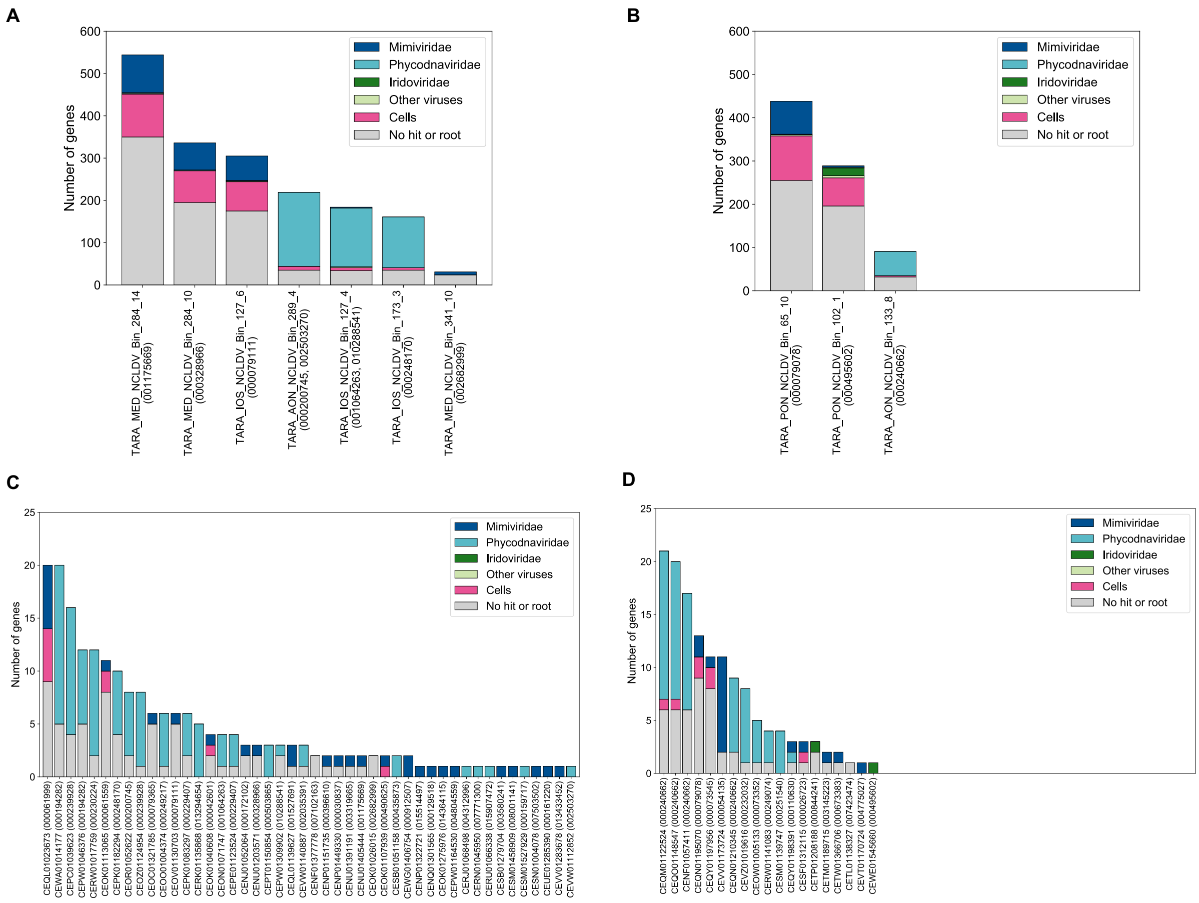

Figure S8. Taxonomic composition of genes predicted in viral genome fragments encoding NCLDV PolBs associated with CEE (VIP score > 2), Related to Table 2. Taxonomic annotations were performed as described in Transparent Methods. (A and B) Metagenome-assembled genomes (MAGs) derived from samples filtered to retain particles of sizes > 0.8 µm encoding PolBs positively (A) or negatively (B) associated with CEE. (C and D) Contigs derived from samples filtered to retain particles between 0.2 µm and 3 µm in size encoding PolBs positively (C) or negatively (D) associated with CEE.

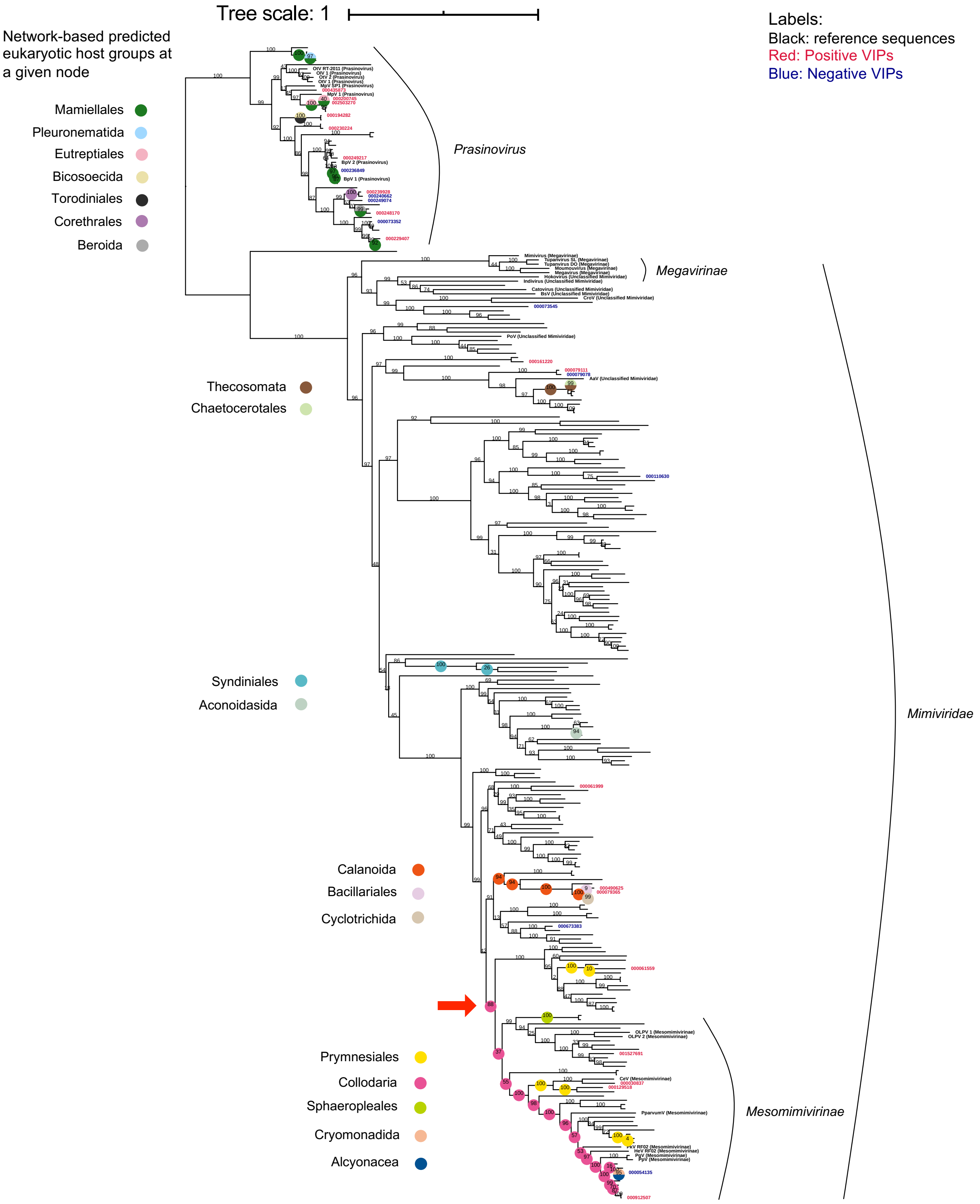

Figure S9: Phylogenetic positions of NCLDV PolBs associated with CEE and network-based predicted eukaryotic host groups, **Related to Table 2; Transparent Methods.** The unrooted maximum likelihood phylogenetic tree contains environmental (labeled in red if VIP score > 2 and the regression coefficient is positive, labeled in blue if negative) and reference (labeled in black) sequences of *Prasinovirus* and *Mimiviridae* PolBs. The approximate SH-like local support values are shown in percentages at nodes, and the scale bar indicates one change per site. Host groups predicted at nodes are shown with colored circles. The red arrow points to a clade of viruses predicted to infect Prymnesiales.

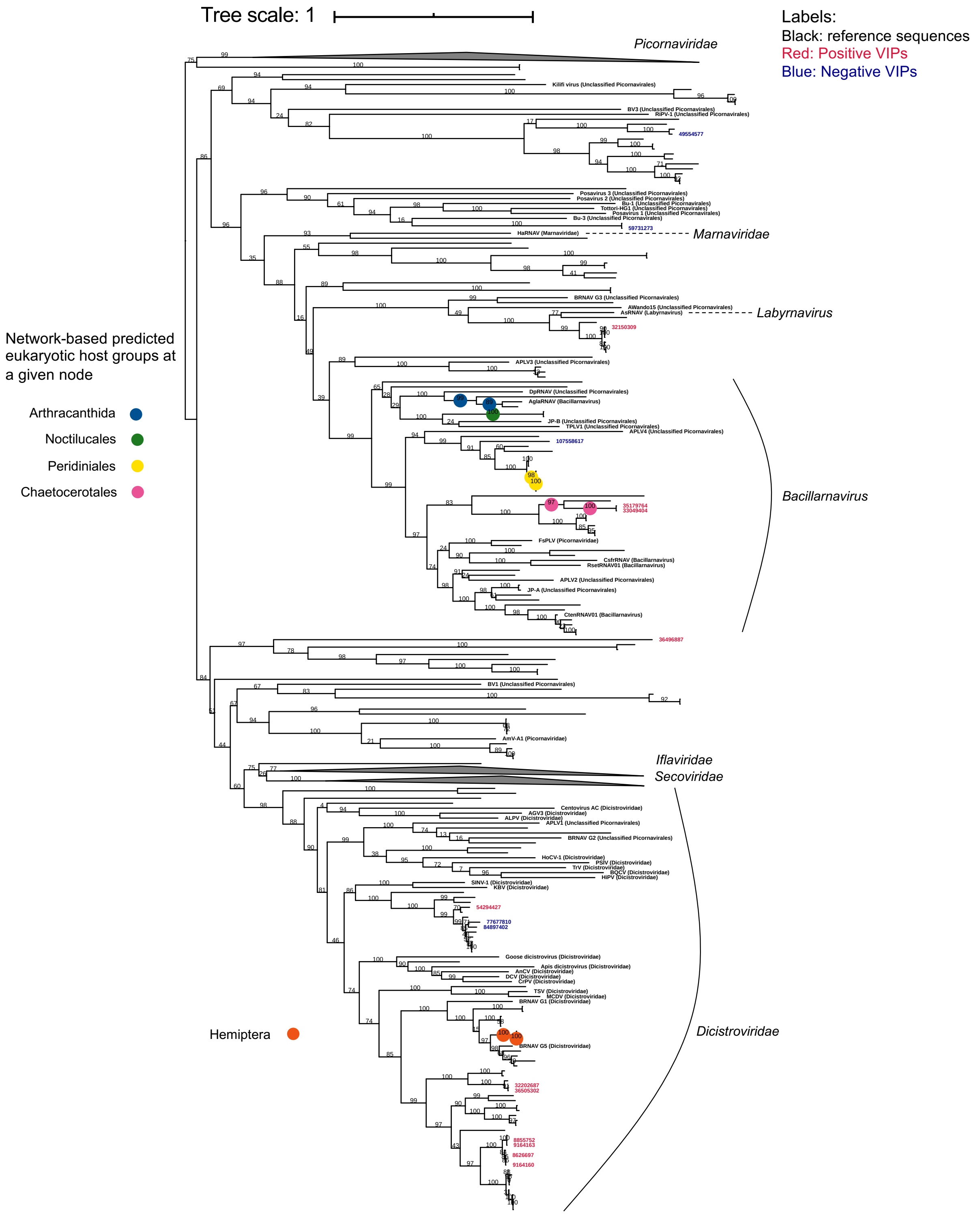

Figure S10: Phylogenetic position of *Piconavirales*s RdRPs associated with CEE and network-based predicted eukaryotic host groups, **Related to Table 2; Transparent Methods.** The unrooted maximum likelihood phylogenetic tree contains environmental (labeled in red if VIP score > 2 and the regression coefficient is positive, labeled in blue if negative) and reference (labeled in black) sequences of *Piconavirale*s RdRPs. The approximate SH-like local support values are shown in percentages at nodes, and the scale bar indicates one change per site. Host groups predicted at nodes are shown with colored circles.

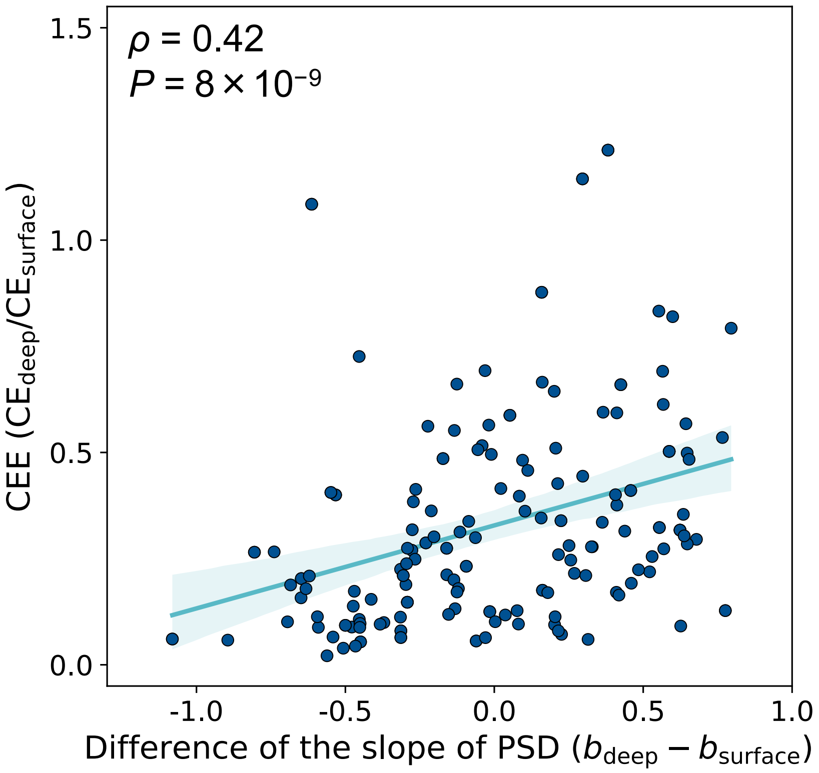

Figure S11. Carbon export efficiency (CEE) is correlated with the change in the slope of particle size distribution (PSD) that occurred from the surface to deep (below the euphotic zone), Rlated to Figure 2A. Observed PSDs were fitted in the form $n = ad^b$, where n is the frequency of particles of a given size, d is the particle diameter, and a and b are parameters (as described by(Guidi et al., 2008)). b, the PSD slope, is a proxy for particles size. For example, b = -5 indicates presence of a large proportion of smaller particles, whereas b = -3 indicates a preponderance of larger particles. A higher b value at deep compared to surface is suggestive of aggregation or presence of larger organisms at deep compare to surface. The blue line shows the regression line between CEE and the PSD slope difference between surface and deep. The shade around the regression line shows the 95% confidence interval.

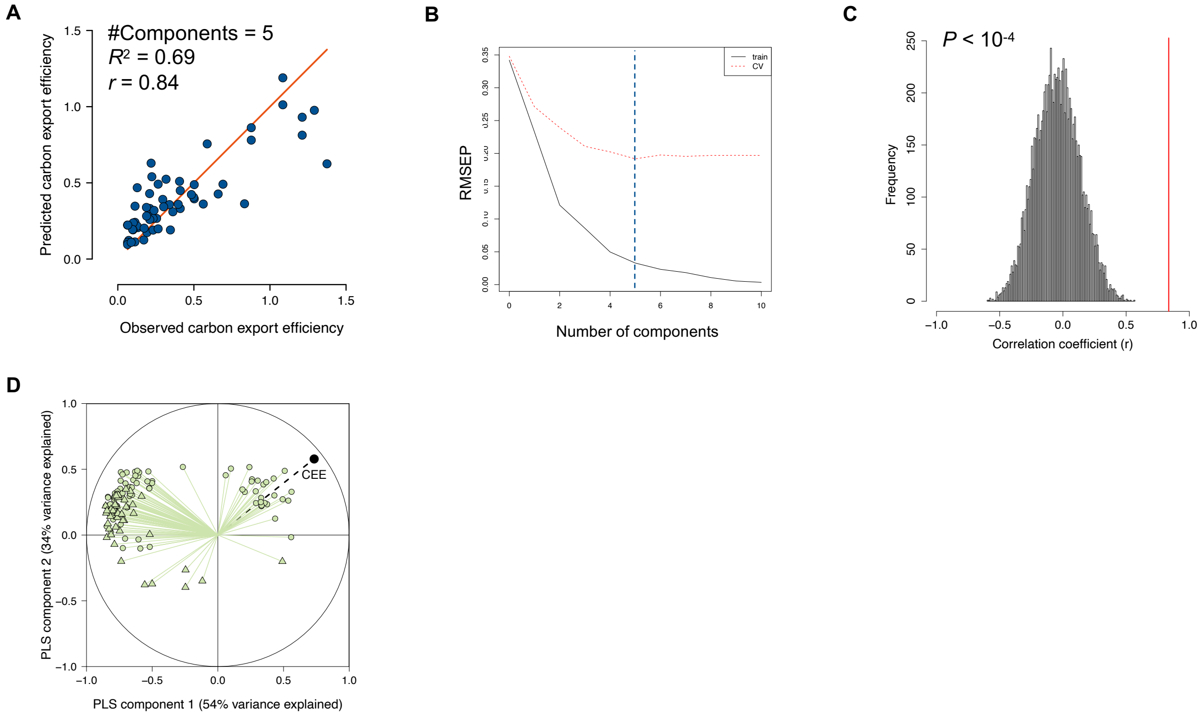

Figure S12. The result of PLS regression using relative abundance profiles of marker-genes of T4-like dsDNA bacteriophages to explain CEE, Related to Figure 3. (A) Bivariate plot between predicted and observed response values in a leave-one-out cross-validation test. (B) Variation in root mean squared error of predictions (RMSEP) across the number of components. (C) Results of the permutation tests (n = 10,000) supporting the significance of the association between viruses and the response variable. (D) Pearson correlation coefficients between the response variable and abundance profiles of viruses with VIP scores > 2 (VIPs) with the first two components in the PLS regression model using all samples. See the legend of Figure S6 for detailed explanation of figures.

Supplemental Tables

Table S1. Viral lineages associated with CEE, Related to Figure 3.

Viruses		VIPs	Positive VIPs	Negative VIPs
NCLDVs	Mimiviridae	34	25	9
	Phycodnaviridae	24	18	6
	Iridoviridae	2	0	2
	Other NCLDVs[a]	0	0	0
	Total	60	43	17
RNA viruses	Picornavirales (ssRNA+)	19	13	6
	Partitiviridae (dsRNA)	1	1	0
	Narnaviridae (ssRNA+)	0	0	0
	Other families	2*	0	2
	Unclassified	0	0	0
	RNA viruses	0	0	0
	Total	22	14	8
ssDNA viruses	Circoviridae	1	1	0
	Geminiviridae	0	0	0
	Nanoviridae	0	0	0
	Unclassified	0	0	0
	ssDNA viruses	0	0	0
	Total	1	1	0
	All	83	58	25

[a]Two Hepeviridae (ssRNA+).

Table S2. Assembly statistics for NCLDV metagenome-assembled genomes and corresponding VIPs, Related to Table 2.

Metagenome-assembled genome	#contigs	N50[a]	L50[b]	Min	Max	Sum	VIPs OTUs (OM-RGC.v1 ID)
TARA_IOS_NCLDV_Bin_127_6	14	21,642	5	8,581	35,822	267,607	PolB 000079111
TARA_IOS_NCLDV_Bin_173_3	12	12,913	3	2,807	34,517	108,412	PolB 000248170
TARA_MED_NCLDV_Bin_284_10	34	10,936	10	2,580	29,722	298,760	PolB 000328966
TARA_MED_NCLDV_Bin_284_14	43	14,837	11	2,756	27,607	439,843	PolB 001175669
TARA_IOS_NCLDV_Bin_127_4	26	5,734	10	2,560	8,505	133,765	PolB 001064263<br>and 010288541
TARA_AON_NCLDV_Bin_289_4	17	9,468	5	3,044	26,201	153,728	PolB 000200745<br>and 002503270
TARA_MED_NCLDV_Bin_341_10	5	7,800	2	2,534	7,941	30,478	PolB 002682999
TARA_PON_NCLDV_Bin_65_10	35	13,866	11	3,781	43,080	382,455	PolB 000079078
TARA_PON_NCLDV_Bin_102_1	53	4,608	18	2,606	11,485	239,832	PolB 000495602
TARA_AON_NCLDV_Bin_133_8	8	7,204	3	2,686	10,349	51,009	PolB 000240662

[a]The length of the contigs for which half of the assembly size is contained in contigs with a length greater than N50.
[b]Number of contigs (or scaffolds) with a size greater or equal to N50.

Table S3. Host prediction per viral OTU for 83 VIPs based on phylogeny, co-occurrence analysis, and genomic context, Related to Table 2.

Virus types	Virus OTUs	Direction of association with CEE	Classification (LCA annotation)	Clade in the trees used for TIM analysis	TIM-based predicted host	MAGs ID	Genome-based predicted host	Suggested host	Note
NCLDVs	polb_000026723	negative	Mimiviridae	NA	NA	NA	NA	NA	
	polb_000030837	positive	Mimiviridae	Mimiviridae/ Mesomimivirinae	Prymnesiales	NA	NA	Prymnesiales	
	polb_000042601	positive	Mimiviridae	NA	NA	NA	NA	NA	
	polb_000054135	negative	Mimiviridae	Mimiviridae/ Mesomimivirinae	Collodaria	NA	NA	Prymnesiales	
	polb_000061559	positive	Mimiviridae	Mimiviridae/ Mesomimivirinae	Prymnesiales	NA	NA	Prymnesiales	
	polb_000061999	positive	Mimiviridae	Mimiviridae	NA	NA	NA	NA	
	polb_000073352	negative	Phycodnaviridae	Phycodnaviridae/ Prasinovirus	NA	NA	NA	Mamiellales	
	polb_000073545	negative	Mimiviridae	Mimiviridae/ CroV relative	NA	NA	NA	NA	
	polb_000079078	negative	Mimiviridae	Mimiviridae/ AaV relative	NA	PON_NCLDV_Bin_65_10	Pelagophyceae	Pelagophycea e	
	polb_000079111	positive	Mimiviridae	Mimiviridae/ AaV relative	NA	IOS_NCLDV_Bin_127_6	Pelagophyceae	Pelagophycea e	
	polb_000079365	positive	Mimiviridae	Mimiviridae	NA	NA	NA	NA	
	polb_000110630	negative	Mimiviridae	Mimiviridae	NA	NA	NA	NA	
	polb_000129518	positive	Mimiviridae	Mimiviridae/ Mesomimivirinae	Prymnesiales	NA	NA	Prymnesiales	
	polb_000159717	positive	Phycodnaviridae	NA	NA	NA	NA	NA	
	polb_000161220	positive	Mimiviridae	Mimiviridae/ AaV relative	NA	NA	NA	Pelagophycea e	
	polb_000172102	positive	Mimiviridae	NA	NA	NA	NA	NA	
	polb_000194282	positive	Phycodnaviridae	Phycodnaviridae/ Prasinovirus	Mamiellales	NA	NA	Mamiellales	
	polb_000200745	positive	Phycodnaviridae	Phycodnaviridae/ Prasinovirus	Mamiellales	AON_NCLDV_Bin_289_4	NA	Mamiellales	
	polb_000229407	positive	Phycodnaviridae	Phycodnaviridae/ Prasinovirus	NA	NA	NA	Mamiellales	
	polb_000230224	positive	Phycodnaviridae	Phycodnaviridae/ Prasinovirus	NA	NA	NA	Mamiellales	
	polb_000232032	negative	Phycodnaviridae	NA	NA	NA	NA	NA	
	polb_000236849	negative	Phycodnaviridae	Phycodnaviridae/ Prasinovirus	Mamiellales	NA	NA	Mamiellales	
	polb_000239928	positive	Phycodnaviridae	Phycodnaviridae/ Prasinovirus	NA	NA	NA	Mamiellales	
	polb_000240662	negative	Phycodnaviridae	Phycodnaviridae/ Prasinovirus	NA	NA	NA	Mamiellales	
	polb_000248170	positive	Phycodnaviridae	Phycodnaviridae/ Prasinovirus	Mamiellales	IOS_NCLDV_Bin_173_3	NA	Mamiellales	
	polb_000249074	negative	Phycodnaviridae	Phycodnaviridae/ Prasinovirus	NA	NA	NA	Mamiellales	
	polb_000249217	positive	Phycodnaviridae	Phycodnaviridae/ Prasinovirus	NA	NA	NA	Mamiellales	
	polb_000251540	negative	Phycodnaviridae	NA	NA	NA	NA	NA	
	polb_000328966	positive	Mimiviridae	NA	NA	NCLDV_Bin_284_10	NA	NA	
	polb_000396610	positive	Mimiviridae	NA	NA	NA	NA	NA	
	polb_000435873	positive	Phycodnaviridae	Phycodnaviridae/ Prasinovirus	NA	NA	NA	Mamiellales	
	polb_000490625	positive	Mimiviridae	Mimiviridae	NA	NA	NA	NA	
	polb_000495602	negative	Iridoviridae	NA	NA	NCLDV_Bin_102_1	NA	NA	
	polb_000503865	positive	Phycodnaviridae	NA	NA	NA	NA	NA	
	polb_000673383	negative	Mimiviridae	Mimiviridae	NA	NA	NA	NA	
	polb_000844241	negative	Iridoviridae	NA	NA	NA	NA	NA	
	polb_000912507	positive	Mimiviridae	Mimiviridae/ Mesomimivirinae	Collodaria	NA	NA	Prymnesiales	
	polb_001064263	positive	Phycodnaviridae	NA	NA	IOS_NCLDV_Bin_127_4	Mamiellales	Mamiellales	
	polb_001175669	positive	Mimiviridae	NA	NA	MED_NCLDV_Bin_284_14	NA	NA	
	polb_001527691	positive	Mimiviridae	Mimiviridae/ Mesomimivirinae	NA	NA	NA	Prymnesiales	
	polb_002035391	positive	Phycodnaviridae	NA	NA	NA	NA	NA	
	polb_002503270	positive	Phycodnaviridae	Phycodnaviridae/ Prasinovirus	Mamiellales	AON_NCLDV_Bin_289_4	NA	Mamiellales	
	polb_002682999	positive	Mimiviridae	NA	NA	NA	NA	NA	
	polb_003145223	negative	Mimiviridae	NA	NA	NA	NA	NA	
	polb_003319665	positive	Mimiviridae	NA	NA	NA	NA	NA	
	polb_003580241	positive	Mimiviridae	NA	NA	NA	NA	NA	

	polb_004312996	positive	Phycodnaviridae	NA	NA	NA	NA	NA	
	polb_004775027	negative	Mimiviridae	NA	NA	NA	NA	NA	
	polb_004804559	positive	Mimiviridae	NA	NA	NA	NA	NA	
	polb_007102163	positive	Mimiviridae	NA	NA	NA	NA	NA	
	polb_007423474	negative	Mimiviridae	NA	NA	NA	NA	NA	
	polb_007503502	positive	Mimiviridae	NA	NA	NA	NA	NA	
	polb_007771300	positive	Phycodnaviridae	NA	NA	NA	NA	NA	
	polb_008001141	positive	Mimiviridae	NA	NA	NA	NA	NA	
	polb_010288541	positive	Phycodnaviridae	NA	NA	IOS_NCLDV_Bin_127_4	Mamiellales	Mamiellales	
	polb_013294654	positive	Phycodnaviridae	NA	NA	NA	NA	NA	
	polb_013433452	positive	Mimiviridae	NA	NA	NA	NA	NA	
	polb_014364115	positive	Mimiviridae	NA	NA	NA	NA	NA	
	polb_015514497	positive	Mimiviridae	NA	NA	NA	NA	NA	
	polb_015907472	positive	Phycodnaviridae	NA	NA	NA	NA	NA	
RNA viruses	rdrp_105714054	negative	Picornavirales	NA	NA	NA	NA	NA	
	rdrp_107558617	negative	Picornavirales	Picornavirales/ Bacillarnavirus	NA	NA	NA	NA	
	rdrp_30787766	positive	Picornavirales	NA	NA	NA	NA	NA	
	rdrp_32150057	positive	Picornavirales	NA	NA	NA	NA	NA	
	rdrp_32150309	positive	Picornavirales	Picornavirales/ Labyrnavirus	NA	NA	NA	Labyrinthulom ycetes	a
	rdrp_32202687	positive	Picornavirales	Picornavirales/ Dicistroviridae	NA	NA	NA	Copepoda	b
	rdrp_33049404	positive	Picornavirales	Picornavirales/ Bacillarnavirus	Chaetocerotal es	NA	NA	Chaetocerotal es	
	rdrp_35179764	positive	Picornavirales	Picornavirales/ Bacillarnavirus	Chaetocerotal es	NA	NA	Chaetocerotal es	
	rdrp_35713768	positive	Partitiviridae	NA	NA	NA	NA	NA	
	rdrp_36496887	positive	Picornavirales	Picornavirales	NA	NA	NA	NA	
	rdrp_36505302	positive	Picornavirales	Picornavirales/ Dicistroviridae	NA	NA	NA	Copepoda	b
	rdrp_42335229	negative	Hepeviridae	NA	NA	NA	NA	NA	
	rdrp_49554577	negative	Picornavirales	Picornavirales	NA	NA	NA	NA	
	rdrp_54294427	positive	Picornavirales	Picornavirales/ Dicistroviridae	NA	NA	NA	Copepoda	b
	rdrp_59731273	negative	Picornavirales	Picornavirales	NA	NA	NA	NA	
	rdrp_77677770	negative	Hepeviridae	NA	NA	NA	NA	NA	
	rdrp_77677810	negative	Picornavirales	Picornavirales/ Dicistroviridae	NA	NA	NA	Copepoda	b
	rdrp_84897402	negative	Picornavirales	Picornavirales/ Dicistroviridae	NA	NA	NA	Copepoda	b
	rdrp_8626697	positive	Picornavirales	Picornavirales/ Dicistroviridae	NA	NA	NA	Copepoda	b
	rdrp_8855752	positive	Picornavirales	Picornavirales/ Dicistroviridae	NA	NA	NA	Copepoda	b
	rdrp_9164160	positive	Picornavirales	Picornavirales/ Dicistroviridae	NA	NA	NA	Copepoda	b
	rdrp_9164163	positive	Picornavirales	Picornavirales/ Dicistroviridae	NA	NA	NA	Copepoda	b
ssDNA viruses	rep_38177659	positive	Circoviridae	NA	NA	NA	NA	Copepoda	c

[a]This virus was located in well-separated clade containing Aurantiochytrium single-stranded RNA virus (AsRNAV) which is known to infect Labyrinthulomycetes.

[b]These viruses were grouped within Dicistroviridae (known to infect insects) and may therefore infect marine arthropods such as copepods.

[c]This virus was connected with a copepod, mollusk and Collodaria OTUs in the co-occurrence network reconstructed for the mesoplankton size. Circoviridae-like viruses are known to infect copepod.

Table S4. Statistics for the FlashWeave co-occurrence graphs, Related to Table 3; Transparent Methods.

Viral marker gene	Planktonic size fraction[a]	#Samples	#Viral OTUs	#Eukaryotic OTUs	#Edges in graph	#Virus-to-eukaryote edges	#Viruses connected to a eukaryote (%)
NCLDVs PolB	Piconano	99	2269	4936	20934	3594	1735 (76)
	Nano	51	1775	1872	6704	1027	721 (41)
	Micro	92	2205	2524	12189	2101	1299 (59)
	Meso	95	2238	2250	11624	1796	1126 (50)
RNA viruses RdRP	Piconano	60	125	4484	10754	446	122 (98)
	Nano	36	53	1768	2659	124	46 (87)
	Micro	62	124	2407	5351	367	117 (94)
	Meso	62	48	2100	4329	116	42 (88)
ssDNA viruses Rep	Piconano	60	64	4484	10577	205	63 (98%)
	Nano	36	1	1768	2563	2	1 (100%)
	Micro	62	4	2407	5086	9	4 (100%)
	Meso	62	8	2100	4242	24	8 (100%)

[a]Pico: 0.8 to 5 µm, Nano: 5 to 20 µm, Micro: 20 to 180 µm, Meso: 180 to 2000 µm

Table S5: Functional differences between eukaryotes found to be best connected to negative VIPs and non-VIPs, Related to Table 3.

Functional trait	Negative VIPs (*n* = 21)		Non-VIPs (*n* = 983)		*P*-value (Fisher's exact test, two sided)	Adjusted *P*-value (BH) (*Q*)
	Presence	Absence	Presence	Absence		
Chloroplast	3	17	276	690	0.218	0.655
Silicification	0	21	60	920	0.632	0.947
Calcification	0	21	30	950	1.000	1.000

Table S6: Functional differences between eukaryotes found to be best connected to positive and negative VIPs, Related to Table 3.

Functional trait	Positive VIPs (n = 50)		Negative VIPs (n = 21)		*P*-value (Fisher's exact test, two sided)	Adjusted *P*-value (BH) (*Q*)
	Presence	Absence	Presence	Absence		
Chloroplast	20	30	3	17	0.053	0.079
Silicification	11	39	0	21	0.027	0.080
Calcification	1	49	0	21	1.000	1.000

Transparent Methods

Data context

We used publicly available data generated in the framework of the *Tara* Oceans expedition. Single-copy marker-gene sequences for NCLDVs and RNA viruses were identified from two gene catalogs: the Ocean Microbial Reference Gene Catalog (OM-RGC) and the Marine Atlas of *Tara* Oceans Unigenes (MATOU). The viral marker-gene read count profiles used in our study are as previously reported for prokaryotic-sized metagenomes (size fraction 0.2–3 μm) (Sunagawa et al., 2015) and eukaryotic-sized metatranscriptomes (Carradec et al., 2018). Eukaryotic plankton samples (the same samples were used for metatranscriptomes, metagenomes and 18S rRNA V9 meta-barcodes) were filtered for categorization into the following size classes: piconano (0.8–5 μm), nano (5–20 μm), micro (20–180 μm), and meso (180–2,000 μm). Eukaryotic 18S rRNA V9 meta-barcodes used in this study (Ibarbalz et al., 2019) included functional trait annotations (chloroplast-bearing, silicified, and calcified organisms) based on a literature survey. These functionally annotated sequences are available from Zenodo (Henry et al., 2019). Indirect measurements of carbon export (mg m^{-2} d^{-1}) in 5-m increments from the surface to a 1,000-m depth were taken from Guidi et al. (Guidi et al., 2016) The original measurements were derived from the distribution of particle sizes and abundances collected using an underwater vision profiler. These raw data are available from PANGEA (Picheral et al., 2014). Net primary production (NPP) data were extracted and averaged from 8-day composites of the vertically generalized production model (VGPM) (Behrenfeld and Falkowski, 1997) for the week of sampling. Thus, in this study, the comparisons between NPP and other parameters were not made at the same time point. This might have affected the results of the regression analysis, especially if there were any short-term massive bloom events, although there was no bloom signal during the sampling period.

Carbon export, carbon export efficiency, and particle size distribution

Carbon flux profiles (mg m^{-2} d^{-1}) were estimated based on particle size distributions and abundances. The method used for carbon flux estimation was previously calibrated comparing sediment trap measurement and data from imaging instruments (Guidi et al., 2008). Carbon flux values from depths of 30 to 970 meters were divided into 20-m bins, each obtained by averaging the carbon flux values from the designated 20 m in profiles gathered during biological sampling within a 25-km radius over 24 h when less than 50% of data were missing (Figure S5). Carbon export (CE) was defined as the carbon flux at 150 m (Guidi et al., 2016). Carbon export efficiency was calculated as follows: $CEE = CE_{deep}/CE_{surface}$. To compare stations with different water column structures, we defined $CE_{surface}$ as the maximum CE (in a 20 m window) within the first 150 m. CE_{deep} is the average CE (also in a 20 m window) 200 m below this maximum. The 150 m limit serves as a reference point to automatize the calculation of $CE_{surface}$ and CE_{deep}. The 150m-depth layer was selected because often used as a reference depth for drifting sediment trap and because most of the deep chlorophyll maximum (DCM) were shallower except at two (stations 98 (175 m) and 100 (180 m)). The maximum $CE_{surface}$ for these two stations was above 150 m. The sampling strategy of *Tara* Oceans was designed to study a variety of marine ecosystems and to target well-defined meso- to large-scale features (based on remote-sensing data). Therefore, this strategy avoided sampling water with important lateral inputs. Nevertheless, the possibility of having locations with potential lateral transport cannot be excluded.

We also calculated an alternative definition of carbon export efficiency relying on euphotic zone depth (T_{100}), which is often used in the analysis of sediment trap/Thotium field data. T_{100} was calculated as CE 100 m below euphotic zone depth (Ez) divided by CE at Ez (Buesseler et al., 2020). Ez was estimated based on the diffuse attenuation coefficient at 490

nm ($K_d(490)$) using the empirical model (Lin et al., 2016). $K_d(490)$ values were extracted from GlobColour monthly mapped product (ftp://ftp.hermes.acri.fr) built using satellite data.

We obtained the particle size distribution (PSD) profiles generated by the *Tara* Oceans expedition and computed the PSD slope at each depth for all profiles. The slope value (denoted "*b*") is used as the descriptor of the particle size distribution as defined in a previous work (Guidi et al., 2009). For example, $b = -5$ indicates the presence of a large proportion of smaller particles, whereas $b = -3$ indicates a preponderance of larger particles. We averaged the slope values at each sampling site in the same way as for carbon export flux.

Identification of viral marker genes from ocean gene catalogs

Viral genes were collected from two gene catalogs: OM-RGC version 1 and MATOU. Sequences in these two gene catalogs are representatives of clusters of environmental sequences (clustered at 95% nucleotide identity). The OM-RGC data were taxonomically re-annotated, with the NCBI reference tree used to determine the last common ancestor modified to reflect the current classification of NCLDVs (Carradec et al., 2018). We automatically classified viral gene sequences as eukaryotic or prokaryotic according to their best BLAST score against viral sequences in the Virus-Host Database (Mihara et al., 2016). DNA polymerase B (PolB), RNA-dependent RNA polymerase (RdRP), replication-associated protein (Rep), and major capsid protein (Gp23) genes were used as markers for NCLDVs, RNA viruses, ssDNA viruses, and T4-like dsDNA bacteriophages, respectively. For PolB, reference proteins from the NCLDV orthologous gene cluster NCVOG0038 (Yutin et al., 2009) were aligned using MAFFT-*linsi* (Katoh and Standley, 2013). A hidden Markov model (HMM) profile was constructed from the resulting alignment using *hmmbuild* (Eddy, 2011). This PolB HMM profile was searched against OM-RGC amino acid sequences and translated MATOU sequences annotated as NCLDVs, and sequences longer than 200 amino acids that had hits with *E*-values $< 1 \times 10^{-5}$ were selected as putative PolBs. RdRP sequences were

chosen from the MATOU catalog as follows: sequences assigned to Pfam profiles PF00680, PF00946, PF00972, PF00978, PF00998, PF02123, PF04196, PF04197, or PF05919 and annotated as RNA viruses were retained as RdRPs. For Rep, we reconstructed an HMM profile using a comprehensive set of reference sequences (Kazlauskas et al., 2018) and searched this profile against the translated MATOU sequences annotated as ssDNA viruses. For Gp23, OM-RGC sequences assigned to Pfam profile PF07068 and annotated as viruses were retained. We kept sequences that had hits with E-values $< 1 \times 10^{-5}$ and removed those that contained frameshifts.

The procedure above identified 3,486 PolB and 6,438 Gp23 sequences in the metagenomic samples and 975 RdRP, 388 PolB, and 299 Rep sequences in the metranscriptomes.

Testing for associations between viruses with CEE, CE_{150}, NPP, and T_{100}

To test for associations between occurrence of viral marker genes and CEE, CE_{150}, NPP, and T_{100} (response variables), we proceeded as follows. Samples with CEE values greater than one and with Z-score greater than two were considered as outliers and removed (this removed the two samples from station 68). Only marker genes represented by at least two reads in five or more samples were retained (lowering this minimal number of required samples down to three or four did not improve the PLS regression model). To cope with the sparsity and composition of the data, gene-length normalized read count matrices were center log-ratio transformed, separately for ssDNA viruses, RNA viruses and NCLDVs. We next selected genes with Spearman correlation coefficients with the response variable greater than 0.2 or smaller than −0.2 (zero values were removed). To assess the association between these marker genes and the response variable, we used partial least square (PLS) regression analysis. The number of components selected for the PLS model was chosen to minimize the root mean square error of prediction (Figures S6 and S7). We assessed the strength of the association

between the response variable and viral marker genes occurrence (the explanatory variables) by correlating leave-one-out cross-validation predicted values with the measured values of the response variable. We tested the significance of the correlation by comparing the original Pearson coefficients between explanatory and response variables with the distribution of Pearson coefficients obtained from PLS models reconstructed based on permutated data (10,000 iterations). We estimated the contribution of each gene (predictor) according to its variable importance in the projection (VIP) score derived from the PLS regression model using all samples. The VIP score of a predictor estimates its contribution in the PLS regression. Predictors with high VIP scores (> 2) were assumed to be important for the PLS prediction of the response variable.

Phylogenetic analysis

Environmental PolB sequences annotated as NCLDVs were searched against reference NCLDV PolB sequences using BLAST. Environmental sequences with hits to a reference sequence that had > 40% identity and an alignment length greater than 400 amino acids were kept and aligned with reference sequences using MAFFT-*linsi*. Environmental RdRP sequences were translated into six frames of amino acid sequences and combined together with reference RNA viruses RdRP sequences collected from the Virus-Host Database. They were searched against the Conserved Domain Database (CDD) using rpsBLAST. The resulting alignment was used to trim reference and environmental RdRP sequences to the conserved part corresponding to the domain, CDD: 279070, before alignment with MAFFT-*linsi*. Rep sequences annotated as ssDNA viruses were treated similarly. PolB, RdRP, and Rep multiple sequence alignments were manually curated to discard poorly aligned sequences. Phylogenetic trees were reconstructed using the the *build* function of ETE3 (Huerta-Cepas et al., 2016) of the GenomeNet TREE tool (https://www.genome.jp/tools-bin/ete). Columns

were automatically trimmed using *trimAl* (Capella-Gutiérrez et al., 2009), and trees were constructed using FastTree with default settings (Price et al., 2009).

A similar procedure was applied for the trees used in the hosts prediction analysis albeit selecting sequences for the Phycodnaviridae/Mimiviridae (Figure S9) and the Picornavirales (Figure S10) and removing the ones occurring in fewer than 10 samples, to reduce the size of the tree.

Virus–eukaryote co-occurrence analysis

We used FlashWeave (Tackmann et al., 2019) with Julia 1.2.0 to predict virus–host interactions based on their co-occurrence patterns. FlashWeave is a novel approach to inferring direct co-occurrence associations based on the local-to-global learning. Read count matrices for the 3,486 PolBs, 975 RdRPs, 299 Reps, and 18S rRNA V9 DNA barcodes obtained from samples collected at the same location were fed into FlashWeave. The 18S rRNA V9 data were filtered to retain OTUs with an informative taxonomic annotation. The 18S rRNAV9 OTUs and viral marker sequences were further filtered to conserve only those present in at least five samples. FlashWeave networks were learned for each of the four eukaryotic size fractions with the parameters ‘heterogenous’ = false and ‘sensitive’ = true, and edges receiving a weight > 0.2 and a Q-value < 0.01 (the default) were retained. The number of samples per size fraction ranged between 51 and 99 for NCLDVs and between 36 and 62 for RNA and ssDNA viruses. The number of retained OTUs per size fraction varied between 1,775 and 2,269 for NCLDVs and between 48 and 125 for RNA viruses (Table S4).

Mapping of putative hosts onto viral phylogenies

In our association networks, individual viral sequences were often associated with multiple 18S rRNA V9 OTUs belonging to drastically different eukaryotic groups, a situation that can reflect interactions among multiple organisms but also noise associated with this type of

analysis (Coenen and Weitz, 2018). To extract meaningful information from these networks, we reasoned as follows. We assumed that evolutionarily related viruses infect evolutionarily related organisms, similar to the case of phycodnaviruses (Clasen and Suttle, 2009). In the interaction networks, the number of connections between viruses in a given clade and the associated eukaryotic host group should accordingly be enriched compared with the number of connections with non-host organisms arising by chance. Following this reasoning, we assigned the most likely eukaryotic host group as follows. The tree constructed from viral marker-gene sequences (PolB, RdRP or Rep) was traversed from root to tips to visit every node. We counted how many connections existed between leaves of each node and the V9-OTUs of a given eukaryotic group (order level). We then tested whether the node was enriched compared with the rest of the tree using Fischer's exact test and applied the Benjamini–Hochberg procedure to control the false discovery rate among comparisons of each eukaryotic taxon (order level). To avoid the appearance of significant associations driven by a few highly connected leaves, we required half of the leaves within a node to be connected to a given eukaryotic group. Significant enrichment of connections between a virus clade and a eukaryotic order was considered to be indicative of a possible virus–host relationship. We refer to the above approach, in which taxon interactions are mapped onto a phylogenetic tree of a target group using the organism's associations predicted from a species co-occurrence-based network, as TIM, for Taxon Interaction Mapper. This tool is available at https://github.com/RomainBlancMathieu/TIM. This approach can be extended to interactions other than virus–host relationships. It has been shown that TIM filtering improves the performance of network-based host prediction for NCLDVs in a benchmark study (Meng et al. (2020). bioRxiv https://doi.org/10.1101/2020.10.16.342030).

Assembly of NCLDV metagenome-assembled genomes (MAGs)

NCLDV metagenome-assembled genomes (MAGs) were assembled from *Tara* Oceans metagenomes corresponding to size fractions > 0.8 μm. Metagenomes were first organized into 11 'metagenomic sets' based upon their geographic coordinates, and each set was co-assembled using MEGAHIT (Li et al., 2015) v.1.1.1. For each set, scaffolds longer than 2.5 kbp were processed within the bioinformatics platform anvi'o (Eren et al., 2015) v.6.1 following methodology described previously for genome-resolved metagenomics (Delmont et al., 2018). Briefly, we used the automatic binning algorithm CONCOCT (Alneberg et al., 2014) to identify large clusters of contigs using both sequence composition and differential coverage across metagenomes within the set. We then used HMMER (Eddy, 2011) v3.1b2 to search for a collection of eight NCLDV gene markers (Guglielmini et al., 2019), and identified NCLDV MAGs by manually binning CONCOCT clusters of interest using the anvi'o interactive interface. The interface displayed hits for the eight gene markers alongside coverage values across metagenomes and GC-content. Finally, NCLDV MAGs were manually curated using the same interface, to minimize contamination as described previously (Delmont and Eren, 2016).

Taxonomic composition of genes predicted in NCLDV genomes of VIPs

VIP's PolB sequences were searched (using BLAST) against MAGs reconstructed from the metagenomes of the eukaryotic size fraction (> 0.8 μm) and against contigs used to produce OM-RGCv1. Genome fragments covering 95% of the length of PolB VIPs with > 95% nucleotide identity were considered as originating from a same viral OTUs. Genes were predicted and annotated taxonomically with the same procedure described above (identification of viral marker genes). Genes contained in viral genome fragments and annotated as cellular organisms with amino acid identities > 60% were manually inspected (Supplemental Data 2).

Statistical test

All the statistical significance assessments were performed with two-sided test.

Supplemental References

Alneberg, J., Bjarnason, B.S., Bruijn, I. de, Schirmer, M., Quick, J., Ijaz, U.Z., Lahti, L., Loman, N.J., Andersson, A.F., and Quince, C. (2014). Binning metagenomic contigs by coverage and composition. Nat. Methods *11*, 1144–1146.

Behrenfeld, M.J., and Falkowski, P.G. (1997). Photosynthetic rates derived from satellite-based chlorophyll concentration. Limnol. Oceanogr. *42*, 1–20.

Buesseler, K.O., Boyd, P.W., Black, E.E., and Siegel, D.A. (2020). Metrics that matter for assessing the ocean biological carbon pump. Proc. Natl. Acad. Sci. *117*, 9679–9687.

Capella-Gutiérrez, S., Silla-Martínez, J.M., and Gabaldón, T. (2009). trimAl: a tool for automated alignment trimming in large-scale phylogenetic analyses. Bioinforma. Oxf. Engl. *25*, 1972–1973.

Carradec, Q., Pelletier, E., Silva, C.D., Alberti, A., Seeleuthner, Y., Blanc-Mathieu, R., Lima-Mendez, G., Rocha, F., Tirichine, L., Labadie, K., et al. (2018). A global ocean atlas of eukaryotic genes. Nat. Commun. *9*, 373.

Clasen, J.L., and Suttle, C.A. (2009). Identification of freshwater Phycodnaviridae and their potential phytoplankton hosts, using DNA pol sequence fragments and a genetic-distance analysis. Appl. Environ. Microbiol. *75*, 991–997.

Coenen, A.R., and Weitz, J.S. (2018). Limitations of Correlation-Based Inference in Complex Virus-Microbe Communities. MSystems *3*, e00084-18.

Delmont, T.O., and Eren, A.M. (2016). Identifying contamination with advanced visualization and analysis practices: metagenomic approaches for eukaryotic genome assemblies. PeerJ *4*, e1839.

Delmont, T.O., Quince, C., Shaiber, A., Esen, Ö.C., Lee, S.T., Rappé, M.S., McLellan, S.L., Lücker, S., and Eren, A.M. (2018). Nitrogen-fixing populations of Planctomycetes and Proteobacteria are abundant in surface ocean metagenomes. Nat. Microbiol. *3*, 804–813.

Eddy, S.R. (2011). Accelerated Profile HMM Searches. PLOS Comput. Biol. *7*, e1002195.

Eren, A.M., Esen, Ö.C., Quince, C., Vineis, J.H., Morrison, H.G., Sogin, M.L., and Delmont, T.O. (2015). Anvi'o: an advanced analysis and visualization platform for 'omics data. PeerJ *3*, e1319.

Guglielmini, J., Woo, A.C., Krupovic, M., Forterre, P., and Gaia, M. (2019). Diversification of giant and large eukaryotic dsDNA viruses predated the origin of modern eukaryotes. Proc. Natl. Acad. Sci. *116*, 19585–19592.

Guidi, L., Jackson, G.A., Stemmann, L., Miquel, J.C., Picheral, M., and Gorsky, G. (2008). Relationship between particle size distribution and flux in the mesopelagic zone. Deep Sea Res. Part Oceanogr. Res. Pap. *55*, 1364–1374.

Guidi, L., Stemmann, L., Jackson, G.A., Ibanez, F., Claustre, H., Legendre, L., Picheral, M., and Gorskya, G. (2009). Effects of phytoplankton community on production, size, and export of large aggregates: A world-ocean analysis. Limnol. Oceanogr. *54*, 1951–1963.

Guidi, L., Chaffron, S., Bittner, L., Eveillard, D., Larhlimi, A., Roux, S., Darzi, Y., Audic, S., Berline, L., Brum, J.R., et al. (2016). Plankton networks driving carbon export in the oligotrophic ocean. Nature *532*, 465.

Henry, N., de Vargas, C., Audic, S., Tara Oceans Consortium, C., and Tara Oceans Expedition, P. (2019). Total V9 rDNA information organized at the OTU level for the Tara Oceans Expedition (2009-2013), including the Tara Polar Circle Expedition (2013). Zenodo https://doi.org/10.5281/zenodo.3768510

Huerta-Cepas, J., Serra, F., and Bork, P. (2016). ETE 3: Reconstruction, Analysis, and Visualization of Phylogenomic Data. Mol. Biol. Evol. *33*, 1635–1638.

Ibarbalz, F.M., Henry, N., Brandão, M.C., Martini, S., Busseni, G., Byrne, H., Coelho, L.P., Endo, H., Gasol, J.M., Gregory, A.C., et al. (2019). Global Trends in Marine Plankton Diversity across Kingdoms of Life. Cell *179*, 1084-1097.e21.

Katoh, K., and Standley, D.M. (2013). MAFFT multiple sequence alignment software version 7: improvements in performance and usability. Mol. Biol. Evol. *30*, 772–780.

Kazlauskas, D., Varsani, A., and Krupovic, M. (2018). Pervasive Chimerism in the Replication-Associated Proteins of Uncultured Single-Stranded DNA Viruses. Viruses *10*, 187.

Li, D., Liu, C.-M., Luo, R., Sadakane, K., and Lam, T.-W. (2015). MEGAHIT: an ultra-fast single-node solution for large and complex metagenomics assembly via succinct de Bruijn graph. Bioinforma. Oxf. Engl. *31*, 1674–1676.

Lin, J., Lee, Z., Ondrusek, M., and Kahru, M. (2016). Attenuation coefficient of usable solar radiation of the global oceans. J. Geophys. Res. Oceans *121*, 3228–3236.

Mihara, T., Nishimura, Y., Shimizu, Y., Nishiyama, H., Yoshikawa, G., Uehara, H., Hingamp, P., Goto, S., and Ogata, H. (2016). Linking Virus Genomes with Host Taxonomy. Viruses *8*, 66.

Picheral, M., Searson, S., Taillandier, V., Bricaud, A., Boss, E., Stemmann, L., Gorsky, G., Tara Oceans Consortium, C., and Tara Oceans Expedition, P. (2014). Vertical profiles of environmental parameters measured from physical, optical and imaging sensors during station TARA_080 of the Tara Oceans expedition 2009-2013. PANGAEA https://doi.org/10.1594/PANGAEA.836419

Price, M.N., Dehal, P.S., and Arkin, A.P. (2009). FastTree: Computing Large Minimum Evolution Trees with Profiles instead of a Distance Matrix. Mol. Biol. Evol. *26*, 1641–1650.

Sunagawa, S., Coelho, L.P., Chaffron, S., Kultima, J.R., Labadie, K., Salazar, G., Djahanschiri, B., Zeller, G., Mende, D.R., Alberti, A., et al. (2015). Ocean plankton. Structure and function of the global ocean microbiome. Science *348*, 1261359.

Tackmann, J., Matias Rodrigues, J.F., and von Mering, C. (2019). Rapid Inference of Direct Interactions in Large-Scale Ecological Networks from Heterogeneous Microbial Sequencing Data. Cell Syst. *9*, 286-296.e8.

Yutin, N., Wolf, Y.I., Raoult, D., and Koonin, E.V. (2009). Eukaryotic large nucleo-cytoplasmic DNA viruses: Clusters of orthologous genes and reconstruction of viral genome evolution. Virol. J. *6*, 223.
